# A comparison of formulations and non-linear solvers for computational modelling of semiconductor devices

**DOI:** 10.1007/s00466-024-02578-x

**Published:** 2024-11-23

**Authors:** Sergi Pérez-Escudero, David Codony, Irene Arias, Sonia Fernández-Méndez

**Affiliations:** 1 Laboratori de Càlcul Numèric, Universitat Politècnica de Catalunya (UPC), Barcelona, Spain; 2 Centre Internacional de Mètodes Numèrics en Enginyeria (CIMNE), Barcelona, Spain

**Keywords:** Semiconductor modelling equations, Finite element (FE) method, Convection-diffusion equations, Numerical oscillations, Gummel solver

## Abstract

The drift-diffusion formulation, modelling semiconductor materials in terms of carrier densities and electric potential, is considered together with an alternative formulation in terms of dimensionless logarithmic quantities. Stability of both formulations in presence of sharp variations with a Galerkin Finite Element discretisation is assessed in two realistic problems: a *p*-*n* junction and an *n*-MOSFET device. The robustness with respect to the initial guess and the computational efficiency of the Newton-Raphson and Gummel non-linear solvers are also compared.

## Introduction

According to the band gap energy, dielectric materials can be classified as insulators, semiconductors or conductors. On one side, insulators have a large band gap, which means that the covalent bonds between atoms are very strong and, in consequence, there are no free electrons to participate in the conduction of current. On the other side, conductors have zero band gap, allowing current conduction. In between, semiconductors have an intermediate band gap value so that, when sufficiently high temperatures are reached or an external electric field is applied, vibrations can break the bonds between atoms and free charges appear, enabling current conduction.

This article focuses on the mathematical and computational modelling of semiconductor devices, composed of several semiconductor materials. It involves three main quantities, referred to as state variables: (1) the electron density, which quantifies the amount of electrons in the conduction band, (2) the hole density, referring to the electrons missing in the valence band in relation to a fully populated one, and (3) the electric potential, which characterises the electric field.

Mathematical and computational modelling of semiconductor devices is key in the design and optimisation of transducers and other semiconductor-based technologies, which nowadays tend towards efficiency and miniaturisation. Essentially, there exist two main mathematical models for semiconductors: the drift-diffusion model, also known as the Van-Roosbroeck equations [29], and the hydrodynamic or energy-transport models [11]. Despite the great attention the hydrodynamic model has received in the last decades, due to its accuracy at sub-micron scales, the most popular model in the semiconductor modelling community is the former, which assumes a constant temperature field, yielding reasonable results at low and moderate electric field regimes. It consists of a coupled, non-linear system of second order partial differential equations (PDE) together with some constitutive relations, and initial and boundary conditions.

The numerical solution of the drift-diffusion model has been a field of research for a long time. Starting from the Scharfetter-Gummel strategy for the one dimensional model [31], several alternative numerical strategies have been developed such as Finite Difference schemes [8, 20], Box methods [10], Finite Volume methods [17, 30], or Finite Element (FE) methods [2, 14, 19], which are the focus of this manuscript. However, the numerical treatment of these equations is not straightforward, since the system of PDE involves convection-dominated convection-diffusion-reaction equations with a non-linear coupling. The solution of these equations typically presents sharp variation layers that usually lead to spurious oscillations in the approximate FE solution. Furthermore, when these numerical oscillations appear near a depletion region, the carrier densities may become negative, which is physically unsound. These facts represent a limitation in the applicability of the standard Finite Element method for solving the drift-diffusion equations.

Several approaches for avoiding or alleviating numerical oscillations in FE solutions have been studied in the last decades, such as the adaptive refinement of the computational mesh [6, 17], discontinuous Galerkin methods [4, 5, 18], or stabilisation techniques, including up-winding schemes that introduce artificial diffusion [15, 21] or the use of residual-free bubbles enriching FE approximations [33]. Discontinous Galerkin methods stabilise the approximation through proper definition of numerical fluxes but they can be computationally expensive due to the increased number of degrees of freedom. Conversely, stabilisation techniques are able to provide oscillation-free approximations, but they usually require parametric tuning and sharp fronts, which in some applications are crucial for an accurate approximation of the output of interest, may be slightly smoothed out.

This document focuses on an alternative, parameter-free strategy, based on considering formulations derived from changes of variables that result in smoother state variables, reminiscent of other formulations such as those based on Quasi-Fermi levels [3, 7] or Slotboom variables [3, 6], that keep the sharpness of the fronts. In particular, this manuscript considers a formulation in terms of dimensionless logarithmic variables that guarantees non-negative carrier densities and provides oscillation-free approximations with relatively coarse meshes, in comparison with the drift-diffusion formulation.

The manuscript is structured as follows. In the second section, the drift-diffusion model is recalled and the corresponding formulation in terms of smoother variables is stated. The third section details the weak forms of both formulations, suitable for a FE discretisation that results in an algebraic non-linear system of equations in each case. In the fourth section, different numerical strategies for solving the aforementioned non-linear systems are considered: a monolithic Newton-Raphson solver and three versions of the Gummel non-linear iterative solver [12], which is here also extended to the new proposed formulation. Numerical experiments are reported in the fifth section. Evidence of the expected Finite Element convergence under nested mesh refinement with a synthetic test is shown for both formulations. More importantly, it is shown that the alternative smoother set of variables enables coarser FE meshes providing oscillation-free solutions, hence reducing the computational cost and mesh generation burden. This claim is confirmed through realistic experiments such as a *p*-*n* junction and an *n*-MOSFET device, by comparing the results obtained in both formulations. In addition, aiming for an efficient complete strategy, a quantitative comparison of convergence, efficiency and robustness is performed for the aforementioned non-linear solvers.

## Semiconductor device modelling equations

### Drift-diffusion equations

The drift-diffusion equations consist of a transient, non-linearly coupled system of second order partial differential equations that can be derived from Maxwell’s equations for semiconductors in isothermal conditions [9]. This manuscript focuses on the study of the stationary version of the equations in dark conditions, where carrier generation by illumination is neglected. It is composed of the Gauss Law and two transport equations,

1a $$\begin{aligned}&\displaystyle {\varvec{\nabla }\cdot \varvec{D}} = \displaystyle {q(p-n+\gamma )}, \end{aligned}$$

1b $$\begin{aligned}&\displaystyle {\varvec{\nabla }\cdot \varvec{J}_n} = \displaystyle {qR(n,p)}, \end{aligned}$$

1c $$\begin{aligned}&\displaystyle {\varvec{\nabla }\cdot \varvec{J}_p} = \displaystyle {-qR(n,p)} \end{aligned}$$

in $\Omega \subset {\mathbb {R}}^m, \ m\in \{1,2,3\}$, together with the constitutive relations

2a $$\begin{aligned}&\varvec{D}= \varvec{\epsilon }\cdot \varvec{E}, \end{aligned}$$

2b $$\begin{aligned}&\varvec{J}_n = q\left( {\textbf{D}}_n \cdot \varvec{\nabla }n + n\varvec{\mu }_n \cdot \varvec{E}\right) , \end{aligned}$$

2c $$\begin{aligned}&\varvec{J}_p = -q\left( {\textbf{D}}_p \cdot \varvec{\nabla }p - p\varvec{\mu }_p \cdot \varvec{E}\right) , \end{aligned}$$

2d $$\begin{aligned}&\varvec{E}= -\nabla \phi , \end{aligned}$$

where *q* denotes the electric charge of a single electron, $\phi$ is the electric potential, and *n* and *p* are the electron and hole carrier densities. $\varvec{E}$ is the electric field; $\varvec{D}$ denotes the electric displacement, that depends on the dielectricity tensor $\varvec{\epsilon }$; and $\varvec{J}_n$ and $\varvec{J}_p$ are the electron and hole current densities, whose expressions depend on the carrier diffusion and mobility tensors ${\textbf{D}}_n$, ${\textbf{D}}_p$, $\varvec{\mu }_n$ and $\varvec{\mu }_p$. For the sake of simplicity, isotropic material tensors are considered throughout the article, that is,

$$\begin{aligned} \varvec{\epsilon }= \epsilon \varvec{I}, \ {\textbf{D}}_n = D_n\varvec{I}, \ {\textbf{D}}_p = D_p\varvec{I}, \ \varvec{\mu }_n = \mu _n\varvec{I}, \ \varvec{\mu }_p = \mu _p\varvec{I} , \end{aligned}$$

where $\epsilon$ denotes the electric permittivity of the material and $D_n,\ D_p, \ \mu _n, \ \mu _p$ denote the diffusivity and mobility parameters. Also, we restrict ourselves to non-degenerate semiconductors, where the Einstein relation between mobility and diffusivity parameters holds:

3 $$\begin{aligned} \frac{D_n}{\mu _n} = \frac{D_p}{\mu _p} = V_T = \frac{k_B T}{q}, \end{aligned}$$

where $V_T$ denotes the thermal voltage, expressed in terms of the electron charge, *q*, the Boltzmann constant, $k_B$, and the lattice temperature, *T*.

In presence of impurities in the atomic lattice, where some atoms are replaced by atoms of other elements, additional or fewer free carriers are introduced in the electric configuration of the semiconductor material. The doping function, $\gamma$, expresses the amount of impurities introduced in the device, that is, extra electrons (n-type materials, $\gamma >0$) or fewer electrons (p-type materials, $\gamma <0$) in the electric configuration of the atomic lattice. In this article, the following discontinuous expression is assumed:

$$\begin{aligned} \gamma = \left\{ \begin{array}{cl} N_{D}^+ &  \text {in}~n- \text {type regions,}\\ -N_A^- &  \text {in}~p-\text {type regions}, \end{array}\right. \end{aligned}$$

where $N_D^+ \ge 0$ represents the density of donor impurities, which donate some electrons to the conduction band, and $N_A^- \ge 0$ denotes the density of acceptor impurities, which take an electron from the valence band to form a covalent bond yielding there a hole, i.e. a positive charge.

The function *R* in the transport equations models the carrier recombination processes that take place in the material. Here, the Shockley–Read–Hall theory [13, 32] is adopted, that is,

4 $$\begin{aligned} R(n,p) = \frac{np - n_i^2}{\tau _n(p+n_i) + \tau _p(n+n_i)}, \end{aligned}$$

where $\tau _n$ and $\tau _p$ stand for the electron and hole lifetimes, respectively, and $n_i$ is the intrinsic carrier concentration. In non-degenerate semiconductors, $n_i$ is related to the density of states of the conduction band, $N_C$, and valence band, $N_V$, and the band gap energy, $E_{\text {gap}}$, as

$$\begin{aligned} n_i = \sqrt{N_CN_V}\exp {\left( -\frac{E_{\text {gap}}}{2k_BT}\right) } . \end{aligned}$$

The boundary value problem is completed by considering appropriate boundary conditions. To this end, a partition of the boundary of the domain is considered for each state variable:

$$\begin{aligned} \partial \Omega = \Gamma _N^{\Box } \cup \Gamma _D^{\Box }, \ s.t. \ \Gamma _N^{\Box } \cap \Gamma _D^{\Box } = \emptyset , \ \Box = \phi ,n,p, \end{aligned}$$

where the subindexes *N* and *D* refer to the boundaries where Neumann or Dirichlet boundary conditions are set. Hence, the boundary conditions associated to the drift-diffusion equations (1) are:

5 $$\begin{aligned} \begin{array}{rrrrr} \displaystyle {\phi = s_{\phi }} &  \displaystyle {\text {on}\,\,\Gamma _D^{\phi }} & , \quad \quad &  \displaystyle { \varvec{D}\cdot \varvec{\nu }= g_{\phi }} &  \displaystyle {\text {on}\,\,\Gamma _N^{\phi }}, \\ \displaystyle {n = s_{n}} &  \displaystyle {\text {on}\,\,\Gamma _D^{n}} & ,\quad \quad &  \displaystyle {\varvec{J}_n \cdot \varvec{\nu }= g_{n}} &  \displaystyle {\text {on}\,\, \Gamma _N^{n}},\\ \displaystyle {p = s_{p}} &  \displaystyle {\text {on}\,\,\Gamma _D^{p}} & ,\quad \quad &  \displaystyle {\varvec{J}_p \cdot \varvec{\nu }= g_{p}} &  \displaystyle {\text {on}\,\,\Gamma _N^{p}}, \end{array} \end{aligned}$$

where $\varvec{\nu }$ denotes the outward unit normal vector on $\partial \Omega$ and $s_{\phi },s_n,s_p,g_{\phi },g_n,g_p$ are some given scalar functions.

Only Dirichlet and Neumann boundary conditions are considered because ideal Ohmic contacts are assumed in all numerical examples. The treatment of other boundary conditions, such as Robin boundary conditions [23], could also be incorporated in the formulations following similar reasoning.

#### Remark 1

By introducing Eqs. (2b) and (2c) into (1b) and (1c), respectively, it becomes apparent that the transport equations are of convection-diffusion-reaction type for the carrier densities *n* and *p*, with convection velocities in the direction of the electric field. They can be written as

$$\begin{aligned} D_n \varvec{\Delta }n - \mu _n \varvec{\nabla }n \cdot \varvec{\nabla }\phi - n\varvec{\Delta }\phi&= R(n,p),\\ D_p \varvec{\Delta }p + \mu _p \varvec{\nabla }p \cdot \varvec{\nabla }\phi + p\varvec{\Delta }\phi&= R(n,p). \end{aligned}$$

Realistic applications usually correspond to convection-dominated scenarios and, in such circumstances, sharp variations are encountered in the solutions which, as mentioned in [36], can be fairly characterised by the well-known extrinsic *Debye length*,

$$\begin{aligned} \lambda _D = \sqrt{\frac{\epsilon V_T}{qN}} \text { with } N = \max (N_D^+,N_A^-). \end{aligned}$$

The numerical treatment of the set of PDE (1)–(2) with standard FE involves very fine meshes or stabilised formulations [15, 21, 33] in order to avoid spurious oscillations in numerical approximations of the solution.

### Alternative formulation with smoother variables

As a way to relax the sharpness of the variations in the solution and the consequent stringent mesh requirements, the following change of variables is proposed:

6 $$\begin{aligned} \displaystyle {\bar{\phi } = \frac{\phi }{V_T}, \ {\bar{n}} = \text {ln}\left( \frac{n}{n_i}\right) , \ {\bar{p}} = \text {ln}\left( \frac{p}{n_i}\right) }. \end{aligned}$$

The associated system of PDE, that arises from rewriting the original set of equations (1)–(4) in terms of $\bar{\phi }, \ {\bar{n}}$ and ${\bar{p}}$, is:

7 $$\begin{aligned} \begin{array}{rl} \varvec{\nabla }\cdot \varvec{D}&  =\displaystyle { qn_i\left( e^{{\bar{p}}}-e^{{\bar{n}}} + \frac{\gamma }{n_i}\right) },\\ \varvec{\nabla }\cdot \varvec{J}_n &  = q{\bar{R}}({\bar{n}},{\bar{p}}), \\ \varvec{\nabla }\cdot \varvec{J}_p &  = -q{\bar{R}}({\bar{n}},{\bar{p}}) \end{array} \end{aligned}$$

in $\Omega \subset {\mathbb {R}}^m$, together with the constitutive relations

8 $$\begin{aligned} \begin{array}{rl} \varvec{D}&  = -V_T\varvec{\epsilon }\cdot \varvec{\nabla }\bar{\phi },\\ \varvec{J}_n &  = qn_ie^{{\bar{n}}}\left( D_n\varvec{\nabla }{\bar{n}} - \mu _n V_T\varvec{\nabla }\bar{\phi }\right) ,\\ \varvec{J}_p &  = -qn_ie^{{\bar{p}}}\left( D_p\varvec{\nabla }{\bar{p}} + \mu _p V_T\varvec{\nabla }\bar{\phi }\right) , \end{array} \end{aligned}$$

and

$$\begin{aligned} \displaystyle {{\bar{R}}({\bar{n}},{\bar{p}}) = R(n_ie^{{\bar{n}}},n_ie^{{\bar{p}}}) = n_i \frac{e^{{\bar{n}}+{\bar{p}}}-1}{\tau _n\left( e^{{\bar{p}}}+1\right) + \tau _p\left( e^{{\bar{n}}}+1\right) }}. \end{aligned}$$

With the new set of variables (6), the layers in the solution are expected to be smoother and, therefore, coarser meshes can be used. Furthermore, this set of variables ensures positiveness of the electron and hole density variables, in accordance with their physical meaning.

This change of variables is already considered in [24], only for the charge densities, along with many other possible choices, including the Slotboom variables or the Quasi-Fermi levels. There, it is showed that the choice in Eqn. (6) is the one exhibiting a smaller range of variance in a simple diode, making it friendlier than the other ones. Nevertheless, the resulting formulation, (7) and (8), involves more non-linearities due to the new exponential terms, $e^{{\bar{n}}}$ and $e^{{\bar{p}}}$, and the stronger non-linearities in ${\bar{n}}$ and ${\bar{p}}$ in the current density expressions $\varvec{J}_n$ and $\varvec{J}_p$. Unfortunately, as reported in [24] and confirmed by our numerical experiments, these non-linearities hamper the applicability of the formulation due to severe ill-conditioning of the linear systems in the non-linear solver iterations. The combined approach proposed in [24] to circumvent this limitation consists on using the Slotboom variables to solve the non-linear problem, and formulating the linear problems in terms of (6). Here, another strategy is considered.

It turns out that pre-multiplying the transport equations by $e^{-{\bar{n}}}$ and $e^{-{\bar{p}}}$, respectively, improves the previously mentioned conditioning issue of the formulation. A possible explanation for this improvement is that the current density expressions do not longer depend on exponential terms, and the non-linearity in the recombination terms is not aggravated. Considering suitably normalised electric displacement and current densities

9a $$\begin{aligned} \overline{\varvec{D}}(\bar{\phi })&= \frac{1}{V_T}\varvec{D}(\bar{\phi }) = - \varvec{\epsilon }\cdot \varvec{\nabla }\bar{\phi }, \nonumber \\ \overline{\varvec{J}}_n(\bar{\phi },{\bar{n}})&= \frac{1}{n_iD_n}\varvec{J}_n(\bar{\phi },{\bar{n}}) e^{-{\bar{n}}} = q\left( \varvec{\nabla }{\bar{n}}-\varvec{\nabla }\bar{\phi }\right) , \end{aligned}$$

9b $$\begin{aligned} \overline{\varvec{J}}_p(\bar{\phi },{\bar{p}})&= \frac{1}{n_iD_p}\varvec{J}_p(\bar{\phi },{\bar{p}}) e^{-{\bar{p}}} = -q\left( \varvec{\nabla }{\bar{p}} + \varvec{\nabla }\bar{\phi }\right) , \end{aligned}$$

where the Einstein relation (3) is assumed, and the following normalised recombination terms,

$$\begin{aligned}  &   {\bar{R}}_n({\bar{n}},{\bar{p}}) = \frac{1}{n_iD_n}{\bar{R}}({\bar{n}},{\bar{p}})e^{-{\bar{n}}}, \\  &   {\bar{R}}_p({\bar{n}},{\bar{p}}) = \frac{1}{n_iD_p}{\bar{R}}({\bar{n}},{\bar{p}})e^{-{\bar{p}}}, \end{aligned}$$

the system of PDE (7) can be rewritten as

10a $$\begin{aligned}&\varvec{\nabla }\cdot \overline{\varvec{D}}(\bar{\phi }) = \frac{q n_i}{V_T}\left( e^{{\bar{p}}}-e^{{\bar{n}}}+\frac{\gamma }{n_i}\right) , \end{aligned}$$

10b $$\begin{aligned}&\varvec{\nabla }\cdot \overline{\varvec{J}}_n(\bar{\phi },{\bar{n}}) + \varvec{\nabla }{\bar{n}} \cdot \overline{\varvec{J}}_n(\bar{\phi },{\bar{n}}) = q{\bar{R}}_n({\bar{n}},{\bar{p}}), \end{aligned}$$

10c $$\begin{aligned}&\varvec{\nabla }\cdot \overline{\varvec{J}}_p(\bar{\phi },{\bar{n}}) + \varvec{\nabla }{\bar{p}} \cdot \overline{\varvec{J}}_p(\bar{\phi },{\bar{n}}) = -q{\bar{R}}_p({\bar{n}},{\bar{p}}) . \end{aligned}$$

Formulation (9)–(10), which will be referred to as *logarithmic formulation* from now on, is complemented with the boundary conditions derived from applying the change of variables (6) to the boundary conditions (5). They can be written as11with Dirichlet functions $\displaystyle {s_{\bar{\phi }} = \frac{s_{\phi }}{V_T}}$, $\displaystyle {s_{{\bar{n}}} = \text {ln}\left( \frac{s_n}{n_i}\right) }$ and $\displaystyle {s_{{\bar{p}}} }= \displaystyle { \text {ln}\left( \frac{s_p}{n_i}\right) }$, and functions $g_{\bar{\phi }} = g_{\phi }/V_T$, $g_{{\bar{n}}} = e^{-{\bar{n}}}g_n/(n_iD_n)$ and $g_{{\bar{p}}} = e^{-{\bar{p}}}g_p/(n_iD_p)$. In general, the conditions on $\Gamma _{N}^n$ and $\Gamma _N^p$ introduce additional non-linearities, but they reduce to homogeneous Neumann conditions in the case of homogeneous normal currents ($g_n = 0$ or $g_p = 0$), which is the usual scenario.

All in all, the logarithmic formulation (10) does not suffer the ill-conditioning problems of (7) and its applicability is demonstrated in the numerical experiments in Sect. 5.

#### Remark 2

Replacing the constitutive equations (9a) and (9b) in Eqs. (10b) and (10c), respectively, leads to the following system of PDE

12 $$\begin{aligned}&\varvec{\Delta }({\bar{n}} - \bar{\phi }) + \varvec{\nabla }{\bar{n}} \cdot \varvec{\nabla }\left( {\bar{n}} - \bar{\phi }\right) = \displaystyle {{\bar{R}}_n({\bar{n}},{\bar{p}})} \nonumber \\&\varvec{\Delta }({\bar{p}} + \bar{\phi }) + \varvec{\nabla }{\bar{p}} \cdot \varvec{\nabla }\left( {\bar{p}} + \bar{\phi }\right) = \displaystyle {{\bar{R}}_p({\bar{n}},{\bar{p}})}. \end{aligned}$$

Thus, the logarithmic formulation (10) also involves two non-linear convection-diffusion equations, (12), for ${\bar{n}} - \bar{\phi }$ and ${\bar{p}} + \bar{\phi }$, respectively, which actually correspond to the normalised Quasi-Fermi levels, driven by velocity fields corresponding to the normalised carrier density gradients.

#### Remark 3

The chosen adimensionalisation factors, $V_T$ and $n_i$, are the most natural ones in the case of constant temperature and material tensors. However, if they are not constant within the domain (a device composed of different semiconductor materials, temperature-dependent models, etc), any other characteristic constant values can be chosen, namely $\phi ^*$ and $n^*$. In this case, a logarithmic formulation analogous to (9)–(10) can be derived with the normalised electric displacement and current densities

13 $$\begin{aligned} \overline{\varvec{D}}(\bar{\phi })&= \frac{1}{\phi ^*}\varvec{D}(\bar{\phi }) = -\varvec{\epsilon }\cdot \varvec{\nabla }\bar{\phi },\nonumber \\ \overline{\varvec{J}}_n(\bar{\phi },{\bar{n}})&= \frac{1}{n^*}\varvec{J}_n(\bar{\phi },{\bar{n}})e^{-{\bar{n}}} = q\left( D_n\varvec{\nabla }{\bar{n}} - \mu _n\phi ^*\varvec{\nabla }\bar{\phi } \right) , \nonumber \\ \overline{\varvec{J}}_p(\bar{\phi },{\bar{p}})&= \frac{1}{n^*}\varvec{J}_p(\bar{\phi },{\bar{p}})e^{-{\bar{p}}} = -q\left( D_p\varvec{\nabla }{\bar{p}} + \mu _p\phi ^*\varvec{\nabla }\bar{\phi } \right) , \end{aligned}$$

where the Einstein relation (3) cannot be used to simplify the formulae.

#### Remark 4

In the case of devices presenting an interface, $\Gamma _I$, between two different semiconductor materials, the interface conditions for the drift-diffusion formulation, assuming no interface charge and recombination, are

14 $$\begin{aligned} \llbracket \varvec{D}\cdot \varvec{\nu }\rrbracket = 0, \ \llbracket \varvec{J}_n \cdot \varvec{\nu }\rrbracket = 0, \ \llbracket \varvec{J}_p \cdot \varvec{\nu }\rrbracket = 0 \ \text {on} \ \Gamma _I, \end{aligned}$$

with the jump operator defined as $\llbracket a\varvec{\nu }\rrbracket = a^1\varvec{\nu }^1 + a^2 \varvec{\nu }^2$, where the superscripts refer to the two different materials. For the logarithmic formulation, the natural interface conditions are

$$\begin{aligned} \llbracket \overline{\varvec{D}} \cdot \varvec{\nu }\rrbracket = 0, \ \llbracket \overline{\varvec{J}}_n \cdot \varvec{\nu }\rrbracket = 0, \ \llbracket \overline{\varvec{J}}_p \cdot \varvec{\nu }\rrbracket = 0 \ \text {on} \ \Gamma _I, \end{aligned}$$

which taking into account Eqn. (13) and the fact that both ${\bar{n}}$ and ${\bar{p}}$ are continuous, reduce to the ones stated in Eqn. (14).

## Weak formulation for finite element modelling

This section focuses on the solution of the systems of PDE presented in Sect. 2 by means of the standard Galerkin FE method. Weak problem statements are formulated to that end.

### Drift-diffusion equations

The weak form of the drift-diffusion formulation (1)–(2), with boundary conditions (5) is: find $\phi ,n,p \in H^1(\Omega )$ such that $\phi = \phi _D$ on $\Gamma ^{\phi }_D$, $n = n_D$ on $\Gamma ^n_{D}$, $p = p_D$ on $\Gamma ^p_D$, and

15a $$\begin{aligned} \displaystyle {d(\varphi ,\phi ) - \frac{q}{\epsilon }m(\varphi ,p-n+\gamma ) + \frac{1}{\epsilon }r_{\phi }(\varphi )}&\displaystyle {= 0}, \end{aligned}$$

15b $$\begin{aligned} \displaystyle {D_n d(\zeta ,n) - \mu _n c(\zeta ,\phi ,n) + m(\zeta ,R(n,p))}&\displaystyle {= \frac{1}{q}r_n(\zeta )}, \end{aligned}$$

15c $$\begin{aligned} \displaystyle {D_p d(\eta ,p) + \mu _p c(\eta ,\phi ,p) + m(\eta ,R(n,p))}&\displaystyle {= \displaystyle -\frac{1}{q}r_p(\eta )}, \end{aligned}$$

for all $\varphi ,\zeta ,\eta \in H^1(\Omega )$ such that $\varphi = 0$ on $\Gamma ^{\phi }_D$, $\zeta = 0$ on $\Gamma ^{n}_D$ and $\eta = 0$ on $\Gamma ^p_D$, where

$$\begin{aligned} \begin{array}{lllll} d(u,v) &  := \displaystyle {\int _{\Omega } \varvec{\nabla }u \cdot \varvec{\nabla }v \ d\Omega }, \\ r_{_\Box }(u) &  := \displaystyle {\int _{\Gamma _{N}^{\Box }} ug_{_\Box } \ dS}, \ \Box =\phi ,n,p,\\ c(u,v,w) &  := \displaystyle {\int _{\Omega } w(\varvec{\nabla }u \cdot \varvec{\nabla }v) \ d\Omega }, \\ m(u,v) &  := \displaystyle {\int _{\Omega } uv \ d\Omega } \ . \end{array} \end{aligned}$$

### Logarithmic formulation

On the other hand, the weak form of the logarithmic formulation (9)–(10), with boundary conditions (11) is: find $\bar{\phi },{\bar{n}},{\bar{p}} \in H^1(\Omega )$ such that $\bar{\phi } = \bar{\phi }_D$ on $\Gamma ^{\phi }_D$, ${\bar{n}} = {\bar{n}}_D$ on $\Gamma ^n_D$, ${\bar{p}} = {\bar{p}}_D$ on $\Gamma ^p_D$, and

16a $$\begin{aligned}&\displaystyle {d(\varphi ,\bar{\phi }) - \frac{qn_i}{\epsilon V_T}m\left( \varphi ,e^{{\bar{p}}}-e^{{\bar{n}}}+\frac{\gamma }{n_i}\right) +\frac{1}{\epsilon }r_{\bar{\phi }}(\varphi )} \displaystyle {= 0} \end{aligned}$$

16b $$\begin{aligned}&\displaystyle {d(\zeta ,{\bar{n}}{-}\bar{\phi }) {-} c({\bar{n}},{\bar{n}}{-}\bar{\phi },\zeta ) {+} m(\zeta ,{\bar{R}}_n({\bar{n}},{\bar{p}}))} {=} \displaystyle {\frac{1}{q}r_{{\bar{n}}}(\zeta ,{\bar{n}})} \end{aligned}$$

16c $$\begin{aligned}&\displaystyle {d(\eta ,{\bar{p}}{+}\bar{\phi }) {-} c({\bar{p}},{\bar{p}}{+}\bar{\phi },\eta ) {+} m(\eta ,{\bar{R}}_p({\bar{n}},{\bar{p}}))} \displaystyle {{=} {-}\frac{1}{q}r_{{\bar{p}}}(\eta ,{\bar{p}})} \ , \end{aligned}$$

for all $\varphi ,\zeta ,\eta \in H^1(\Omega )$ such that $\varphi = 0$ on $\Gamma ^{\phi }_D$, $\zeta = 0$ on $\Gamma ^{n}_D$ and $\eta = 0$ on $\Gamma ^p_D$, where

$$\begin{aligned} r_{\bar{\phi }}(u) := \int _{\Gamma _N^{\phi }} ug_{\bar{\phi }} \ dS, \,\, \displaystyle {r_{\bar{_\Box }}(u,\bar{_\Box })} := \displaystyle {\int _{\Gamma _N^{\Box }} ug_{\bar{_\Box }} \ dS, \ \Box =n, p}. \end{aligned}$$

The FE solution of (15) and (16) ends by splitting the domain $\Omega$ in a set of non-overlapping elements and considering the approximation space of continuous, piecewise polynomials for the discretisation of the weak equations. More information about the FE method can be found in [28].

## Non-linear solvers

The discretisation of the weak form (15) or (16) in a Finite Element space results in a non-linear system of algebraic equations for the nodal values of the state variables. Its solution is addressed here with two different non-linear solvers: (i) a classical monolithic Newton-Raphson solver and (ii) the Gummel method, with three different versions described next.

### Gummel method for the drift-diffusion equations

The Gummel solver [12] is a very popular non-linear solver among the semiconductor modelling community. Aiming to decouple the system of PDE, it consists on a staggered iteration process based on introducing a non-linearity in the Gauss Law equation, by considering the Quasi-Fermi levels as intermediate variables. In each staggered iteration, *k*, the Quasi-Fermi levels are computed, given the approximation $\phi ^k$, $n^k$ and $p^k$, as

17 $$\begin{aligned} \begin{array}{rl} \phi _n^k = \displaystyle {\phi ^k - V_T\text {ln}\left( \frac{n^k}{n_i}\right) }, \quad \phi _p^k = \displaystyle {\phi ^k + V_T\text {ln}\left( \frac{p^k}{n_i}\right) }. \end{array} \end{aligned}$$

Rewriting the Gauss Law (1a) in terms of $\phi _n^k$ and $\phi _p^k$, and regarding them as known constant values, leads to the following non-linear version of the weak form of the Gauss law (15a) where $\phi ^{k+1}$ is the only unknown,

18 $$\begin{aligned}  &   \displaystyle {d(\varphi ,\phi ^{k+1}) - \frac{qn_i}{\epsilon }m\left( \varphi ,e^{\frac{\phi _p^k-\phi ^{k+1}}{V_T}}-e^{\frac{\phi ^{k+1}-\phi _n^k}{V_T}}+\frac{\gamma }{n_i}\right) }\nonumber \\    &   \quad \displaystyle { + \frac{1}{\epsilon }r_{\phi }(\varphi ) = 0 } . \end{aligned}$$

The potential $\phi ^{k+1}$ is obtained from a Newton-Raphson solution of (18), and it is then taken as data in the weak transport Eqs. (15b) and (15c), resulting in a non-linear system of two coupled equations for the charge densities $n^{k+1}$ and $p^{k+1}$. The updated values of $\phi$, *n* and *p* are used as new initial values to repeat the loop until convergence to the numerical solution ($\phi ^h, \ n^h$ and $p^h$). The whole process is illustrated in Algorithm 1.

**Algorithm 1:**
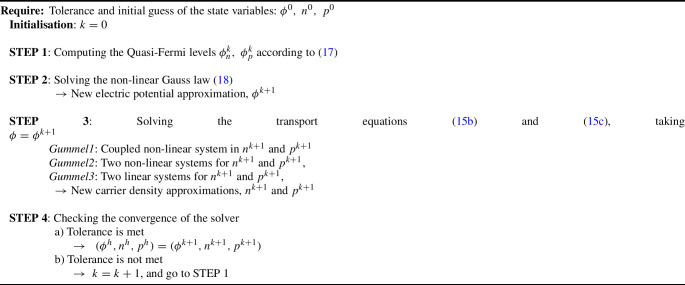
Gummel scheme

The third step in the Gummel method, to compute $n^{k+1}$ and $p^{k+1}$, solution of the transport non-linear system of equations (15b)–(15c), can be solved with a monolithic Newton method, referred to as *Gummel1* in the examples. Another option to reduce the computational cost of this step, labeled as *Gummel2*, is decoupling the system, solving (15b) for $n^{k+1}$ taking $p=p^k$, and solving Eq. (15c) for $p^{k+1}$ taking $n=n^{k}$, leading to two non-linear systems with half size that can be solved with Newton’s method. Finally, one can further reduce the computational cost of the step by regarding the recombination terms as known, with $R(n^k,p^k)$, leading to two decoupled linear systems for $n^{k+1}$ and $p^{k+1}$. Details can be found in Appendix A.

The third option, referred to as *Gummel3* in the examples, is significantly cheaper, but a numerical experiment in Sect. 5 shows how the simplification may disrupt the robustness and the rate of convergence of the Gummel iterations.

### Gummel method for the logarithmic formulation

The Gummel solver can also be adapted to the logarithmic formulation using the expression of the adimensionalised Quasi-Fermi levels

$$\begin{aligned} \begin{array}{rl} \bar{\phi }_n^k = \displaystyle {\bar{\phi }^k-{\bar{n}}^k}, \quad \bar{\phi }_p^k = \displaystyle {\bar{\phi }^k+{\bar{p}}^k} \end{array} \end{aligned}$$

in the first step. In the second step, the updated potential $\bar{\phi }^{k+1}$ is obtained from the non-linear version of the weak form of the Gauss law (16a)

$$\begin{aligned} \displaystyle {d(\varphi ,\bar{\phi }^{k+1}})- &   m\left( \varphi ,\frac{qn_i}{\epsilon V_T}\left( e^{\bar{\phi }_p^k-\bar{\phi }^{k+1}}-e^{\bar{\phi ^{k+1}}-\bar{\phi }_n^k} + \frac{\gamma }{n_i} \right) \right) \\  &   + \frac{1}{\epsilon }r_{\bar{\phi }}(\varphi ) = 0 , \end{aligned}$$

that comes from rewriting the Gauss law (10a) in terms of the Quasi-Fermi levels. The logarithmic formulation also offers the same possibilities in the third step, to compute ${\bar{n}}^{k+1}$ and ${\bar{p}}^{k+1}$ from the transport weak Eqs. (16b) and (16c). Nevertheless, in order to achieve two decoupled linear systems, in addition to considering constant recombination right-hand-side terms, ${\bar{R}}_n({\bar{n}}^k,{\bar{p}}^k), \ {\bar{R}}_p({\bar{n}}^k,{\bar{p}}^k)$, a linearisation of the convective term is also needed. For instance, one can consider as constant the velocity field in the convection term, corresponding to the carrier density gradients (see Remark 2). Details can be found in Appendix A.

#### Remark 5

The linear systems in the Newton iterations, of both the monolithic Newton-Raphson approach and the intermediate steps of the Gummel schemes, have been solved using direct solvers. The Jacobian matrix has roughly the same number of non-zero entries in both formulations, since the additional non-linearities introduced in the logarithmic formulation contribute to the Jacobian matrix in blocks that are also non-zero in the drift-diffusion formulation, without substantially changing its sparsity pattern.

#### Remark 6

It is worth saying that one can consider a large tolerance for the convergence requirements in the intermediate non-linear solutions in steps 1 and 2, or even consider a small fixed number of iterations, still achieving convergence with high accuracy in the Gummel solver, as reported in Sect. 5. This option will be labeled in the experiments as *Gummel1*$^{\clubsuit }$ and *Gummel2*$^{\clubsuit }$.

## Numerical experiments

The applicability and performance of the drift-diffusion and logarithmic formulations is tested in this section through a synthetic example, a *p*-*n* junction and a MOSFET device. The robustness and efficiency of the two non-linear solvers considered in Sect. 4 are also compared in the two first examples.

### Synthetic test

A synthetic test in a square domain $\Omega = (-0.5,0.5)^2$, composed by n-type material for $x<0$ and p-type material for $x>0$, with Dirichlet boundary conditions and the material parameters reported in Table 3 is considered. Uniform quadrilateral meshes are employed and source terms and boundary values are set so that the exact solution is

19 $$\begin{aligned} \begin{array}{ll} \phi (x,y) &  = \sin (x+y)+\cos (x+y)+2, \\ n(x,y) &  = 2\sin (x+y) + \cos (x+y)+3, \\ p(x,y) &  = \sin (x+y)+1 . \end{array} \end{aligned}$$

**Fig. 1:**
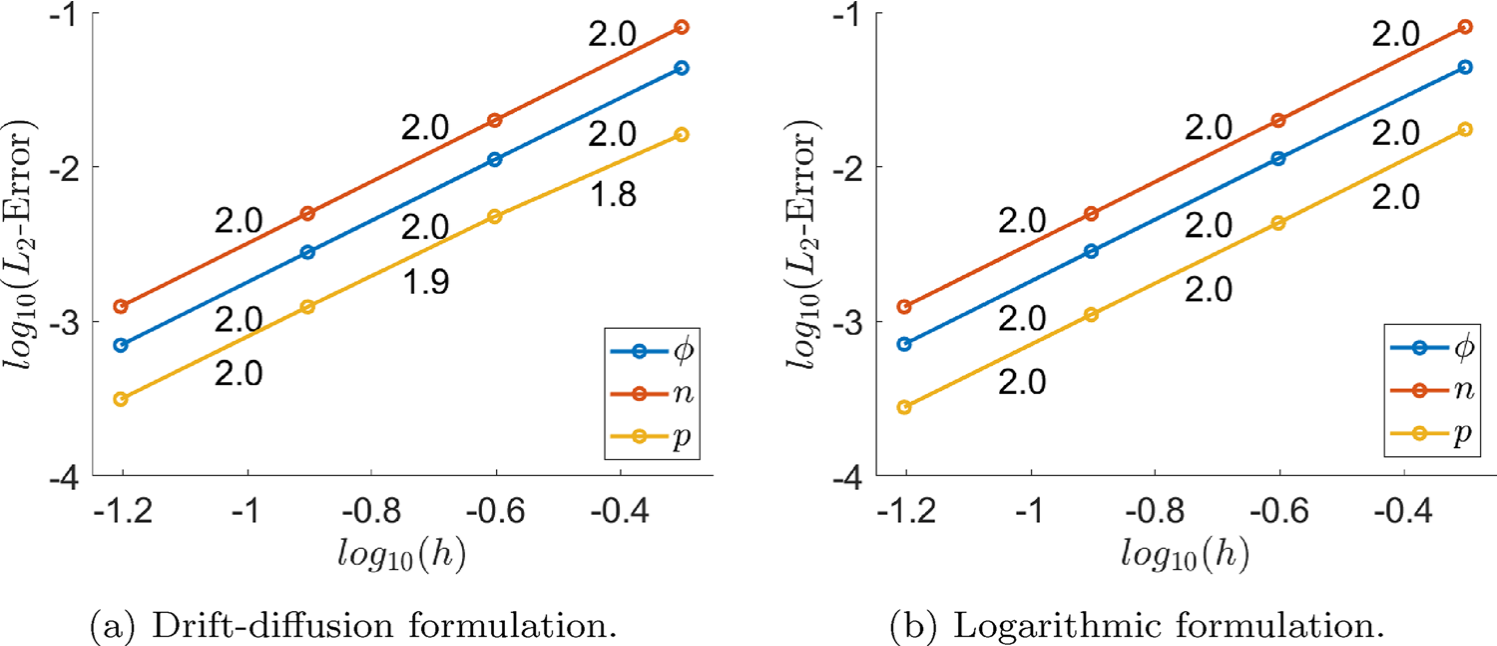
Convergence plots obtained using linear finite elements for each formulation. The numbers in the plots are the slopes of each segment

Figure 1 shows the convergence plot in $L^2$ norm, with linear FE approximation, for the drift-diffusion formulation (left) and for the logarithmic formulation (right), where the expected FE optimal convergence rate can be appreciated for both formulations. In the logarithmic formulation, the error is evaluated for *n* and *p* corresponding to the obtained dimensionless solution ${\bar{n}}$ and ${\bar{p}}$, using the change of variables (6). It can be concluded that both formulations provide similar accuracy for the state variables.

**Fig. 2:**
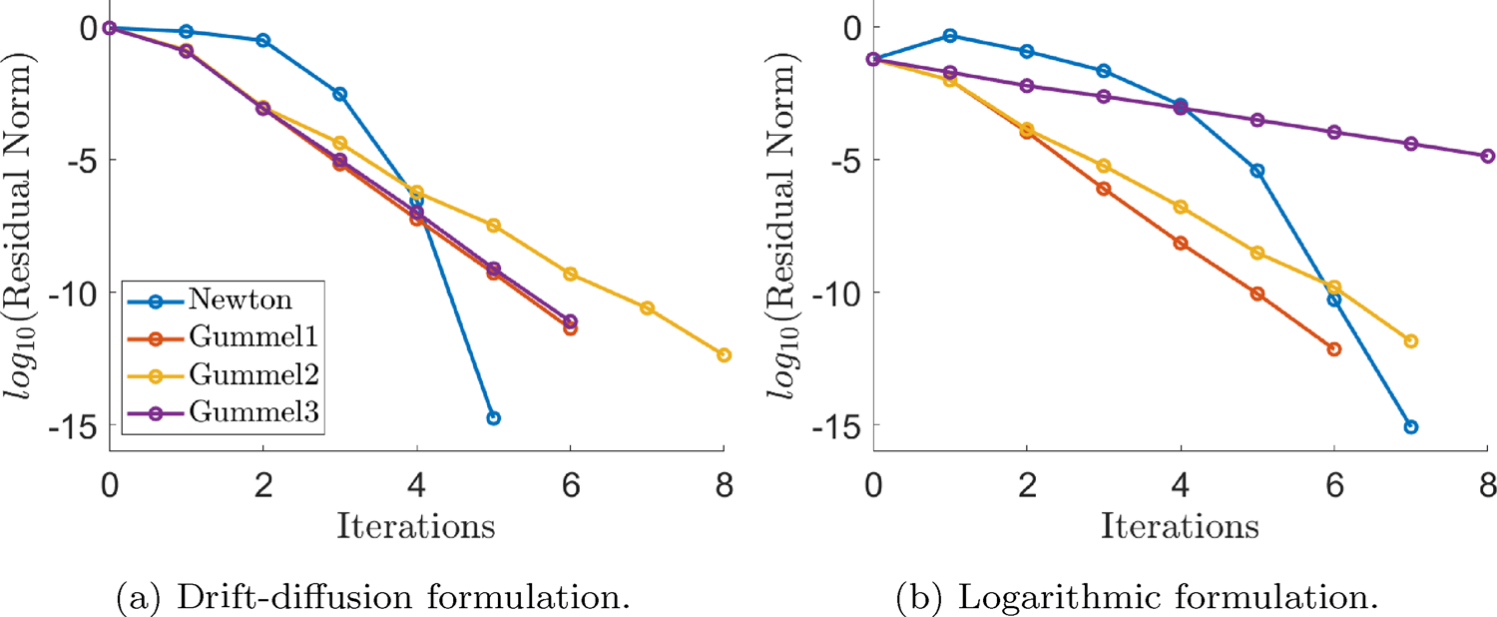
Non-linear solvers convergence plots, for the drift-diffusion formulation with initial guess perturbation $\beta = 5/2$ (left), and for the logarithmic formulation with $\beta =2/3$ (right)

On the other hand, Fig. 2 shows the norm of the residual of the non-linear system corresponding to the discretisation of Eqn. (15) for the drift-diffusion formulation (left) and to the discretisation of Eqn. (16) for the logarithmic formulation (right), divided by the square root of its number of components, for each iteration of the Newton method and the three versions of the Gummel solver. A quadrilateral mesh of element size $h=1/16$ has been used in all cases. The labels *Gummel1*, *Gummel2* and *Gummel3* in the legends correspond to a monolithic Newton solution, two half-size Newton solutions, and two linear system solutions in the third step of the Gummel solver, as explained in Sect. 4. The initial guess is a perturbation of the analytical solution (19), summing $\beta \omega$ to each state variable with

$$\begin{aligned} \omega (x,y) = 30(x{-}0.5)(x{+}0.5)(y{-}0.5)(y+0.5), \ \beta \in {\mathbb {R}}^+. \end{aligned}$$

The expected asymptotic quadratic convergence of Newton-Raphson can be observed for both formulations. Gummel’s method exhibits the expected linear, or almost linear, asymptotic convergence as well, but it is also very fast, and machine accuracy is reached after few iterations.

It is also worth noting that, in this synthetic test, the three versions of the Gummel solver show similar asymptotic convergence in the drift-diffusion formulation, whereas the cheaper third version is significantly slower than the other options for the logarithmic one. We attribute this slowdown in performance due to the need of keeping constant the drift velocity field for the normalised Quasi-Fermi levels in the transport equations, in order to obtain two decoupled linear systems of equations. In any case, we are more interested in the performance of the non-linear solvers in realistic applications as the *p*-*n* junction considered in the next section.

### $p{-}n$ junction

The robustness of the non-linear solvers with respect to the initial guess and the mesh requirements to provide oscillation-free solutions are analysed next, for both formulations, through a realistic experiment: a *p*-*n* junction.

A *p*-*n* junction is obtained when an *n*-type material is attached to a *p*-type material. Under thermal equilibrium, when both materials interact, their free carriers interplay creating an electric field around the interface, also called depletion region. The corresponding difference of potential across both layers is often referred to as built-in potential, $\phi _{\text {bi}}$. With this unbiased setting, charge diffusivity to the other layer is compensated by an opposite drift driven by the built-in electric field, causing no net currents. Thermal equilibrium can be disrupted by the application of an additional voltage drop, causing a bias, i.e. an imbalance of free carriers, that can be used to generate effective current by connecting the two layer ends with a pair of electrodes and a wire.

**Fig. 3:**
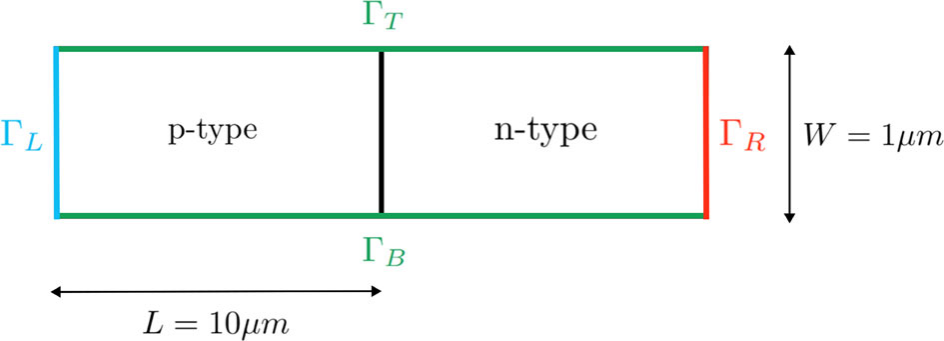
*p*-*n* junction problem statement

A *p*-*n* junction of $20\,\upmu {\textrm{m}}$ length and $1\,\upmu {\textrm{m}}$ width is considered, as depicted in Fig. 3, with two ideal Ohmic contacts located at the left and right boundaries. Assuming that the boundaries are far enough from the depletion region, charge neutrality boundary conditions are considered for the carrier densities on the left and right boundaries of the device, that is,

$$\begin{aligned} n = \frac{N_D^+}{2}\left( 1+ \sqrt{1+4\frac{n_i^2}{{N_D^+}^2}}\right) , \quad p = \frac{n_i^2}{n} \quad&\text {on } \Gamma _{\text {R}}, \\ n = \frac{n_i^2}{p}, \quad p = \frac{N_A^-}{2}\left( 1+\sqrt{1+4\frac{n_i^2}{{N_A^-} ^2}}\right) \quad&\text {on } \Gamma _{\text {L}}. \end{aligned}$$

Zero normal currents, $\varvec{J}_n \cdot \varvec{\nu }= \varvec{J}_p \cdot \varvec{\nu }= 0$, are enforced on $\Gamma _{\text {T}}$ and $\Gamma _{\text {B}}$.

The electric potential Dirichlet boundary conditions impose the difference of potential between both layers, which corresponds to the aforementioned built-in potential minus an applied voltage, $V_{\text {app}}$, that models the effect of an imposed external electric field, that is

$$\begin{aligned}&\phi = 0 \ \text {at} \ \Gamma _{\text {L}}, \ \phi = \phi _{\text {bi}} - V_{\text {app}} \ \text {at} \ \Gamma _{\text {R}}, \text { with }\\  &\phi _{\text {bi}} = V_T\text {ln}\left( \frac{N_D^+N_A^-}{n_i^2}\right) . \end{aligned}$$

At the top and bottom boundaries, $\Gamma _{\text {T}}$ and $\Gamma _{\text {B}}$, zero normal electric displacement is assumed. Note that the homogeneous Neumann boundary conditions of the problem turn this setting into, essentially, a 1D problem along the *p*-*n* junction length dimension. Material parameters used for the simulations are reported in Table 3, which correspond to a Debye length of $\lambda _D \approx 0.31\,\upmu {\textrm{m}}$.

**Fig. 4:**
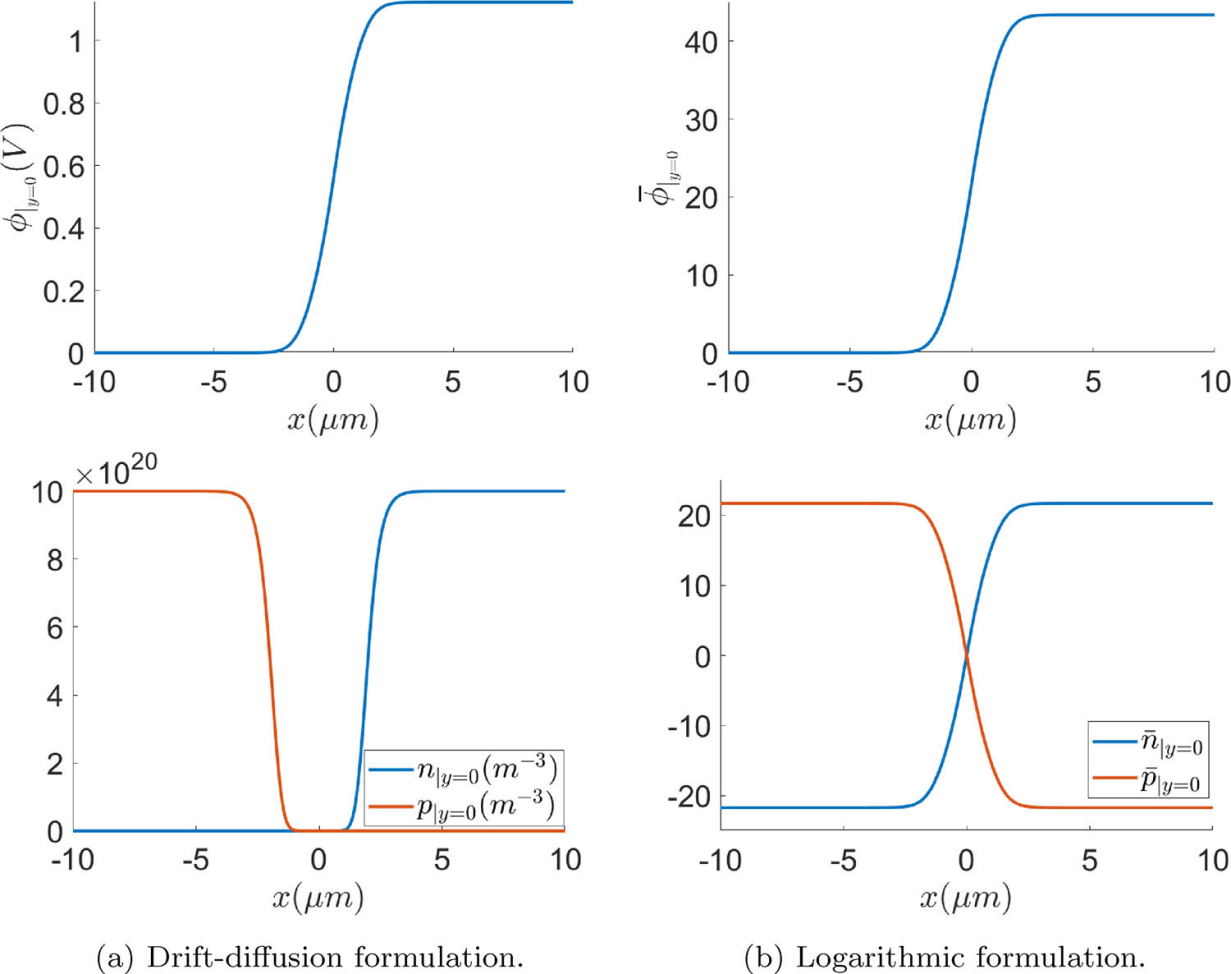
Unbiased ($V_{\text {app}}=0$) *p*-*n* junction solution along the *x*-axis in the middle cross section, with the drift-diffusion (left) and logarithmic (right) formulations

The solution of the unbiased problem ($V_{\text {app}}=0$), computed in 2D with both formulations, using a uniform quadrilateral mesh of $500 \times 4$ elements, is plotted in Fig. 4. The obtained results are physically realistic since the variables are expected to be constant far from the depletion region, while exhibiting sharp variations close to it, as it is reported in [36]. Furthermore, the depletion region is expected to be empty of charges by definition, fact that can also be observed in Fig. 4. It is also worth noting that, as expected, the variation of the state variables for the electron and hole densities is much smoother in the logarithmic formulation.

**Fig. 5:**
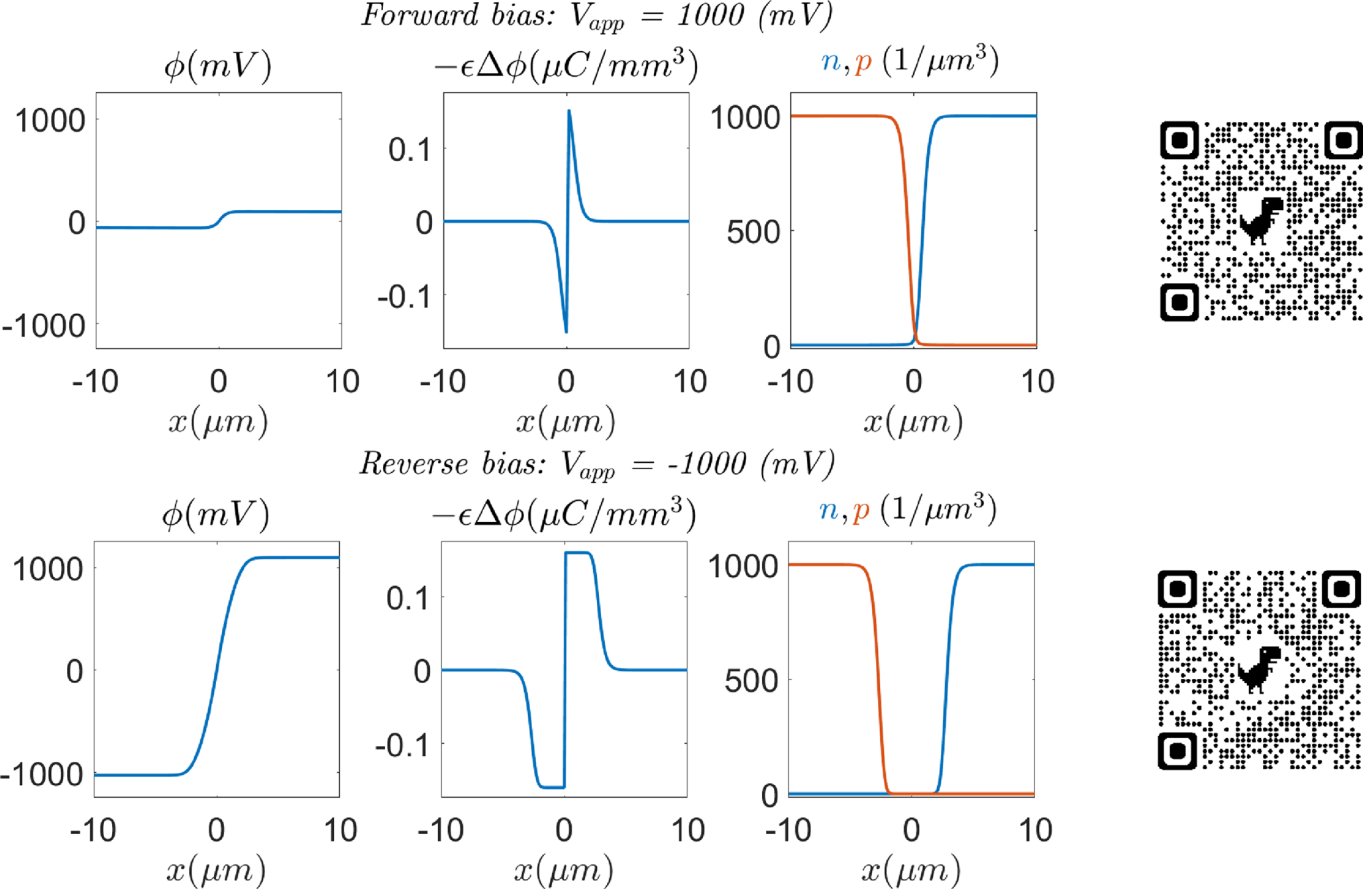
Electric potential (left), electric charge (center) and charge densities (right) obtained in forward (top) and reverse (bottom) bias regimes

Figure 5 shows the electric potential, electric charge and carrier densities in forward and reverse bias regimes with an applied voltage of $V_\text {app} = \pm 1000\,{\textrm{mV}}$, respectively. The Figure provides a comparison between both regimes. It can be appreciated how the difference of potential is larger in the reverse regime, resulting in a wider depletion region, as can be observed in the electric charge plot. Consequently, the charges are more separated. In the forward bias regime, the difference of electric potential is shrunk, so that the electric field is weaker. In consequence, the depletion region is narrower and the charges are closer. Two videos illustrating the behavior of the state variables, currents and recombination terms in forward ($V_{\text {app}}>0$) and reverse ($V_{\text {app}}<0$) bias regimes, computed with the logarithmic formulation, can be found at [26, 27] or scanning the QR codes included in the Figure.

#### Robustness and efficiency comparison of non-linear solvers

The robustness of the non-linear solvers against the initial guess is tested next solving the *p*-*n* junction problem, with $V_{app}=0$, in a uniform FE mesh with $500\times 4$ quadrilateral linear elements and varying the initial guess. The tolerance for the relative error in the solutions and the residual norm is $10^{-8}$ for both the monolithic Newton solver and Gummel’s method. Furthermore, in each iteration of the Gummel method, two different tolerances are considered for the non-linear problems in the second and third steps: $10^{-7}$ to iterate until the intermediate problems are accurately solved, and 1.5 to do fewer iterations for the intermediate problems.

Being $\phi ^h$, $n^h$ and $p^h$ the numerical solutions of the non-linear system of equations up to the aforementioned relative error tolerance, the initial guesses in the next study are defined as

$$\begin{aligned} \phi ^0=\phi ^h+V_T\alpha \omega , \quad n^0=n^h + n_i\alpha \omega , \quad p^0=p^h + n_i\alpha \omega \end{aligned}$$

with $\alpha \in {\mathbb {R}}^+$ and the bubble function

$$\begin{aligned} \omega (x,y) = \frac{3}{80\sqrt{5}}(x-10)(x+10)(y-0.5)(y+0.5), \end{aligned}$$

chosen so that Dirichlet boundary conditions are not affected and the parameter $\alpha$ quantifies the perturbation. The same strategy is applied for the logarithmic formulation, taking as initial guesses

$$\begin{aligned} {\bar{\phi }}^0={\bar{\phi }}^h+\beta \omega , \quad {{\bar{n}}}^0={{\bar{n}}}^h + \beta \omega , \quad {{\bar{p}}}^0={{\bar{p}}}^h + \beta \omega , \end{aligned}$$

with ${\bar{\phi }}^h$, ${{\bar{n}}}^h$ and ${{\bar{p}}}^h$ the solution of the logarithmic non-linear system and $\beta \in {\mathbb {R}}^+$.

Table 1 reports the maximum value for the perturbation parameters, $\alpha$ and $\beta$, providing convergence for each non-linear solver and for both formulations. In the case of the Gummel method, the labels *Gummel1*$^{\clubsuit }$ and *Gummel2*$^{\clubsuit }$ correspond to the relaxed versions with increased tolerance 1.5. Otherwise, the tolerance of $10^{-7}$ has been considered for the intermediate problems in each iteration of the non-linear solver.

No comparison between $\alpha$ and $\beta$ should be made, since the effect of the initial guess perturbation parameter is different for the drift-diffusion state variables and the logarithmic ones.

Analysing each one of the formulations, the *Gummel3* approach, which reduces the third step to linear problems, does not achieve convergence even with initial guesses very close to the solution. This option is consequently discarded in following analyses due to its lack of robustness. On the other hand, it is important highlighting that, depending on the particular problem, a monolithic Newton strategy can be significantly more robust than Gummel’s method, as it is the case for the drift-diffusion formulation here, in contrast with what is claimed in [5, 22].

**Table 1:** Maximum value of the perturbation parameter providing convergence for each non-linear solver and both formulations

	Drift-diffusion ($\alpha$)	Logarithmic ($\beta$)
Newton	$>10^{13}$	1.0980
*Gummel1*	$6.8344\cdot 10$	0.9048
*Gummel1*$^{\clubsuit }$	$2.4394 \cdot 10^2$	0.2459
*Gummel2*	$6.8344\cdot 10$	1.4305
*Gummel2*$^{\clubsuit }$	$2.5998\cdot 10^2$	0.2436
*Gummel3*	$1.2124\cdot 10^{-2}$	$<10^{-6}$

**Fig. 6:**
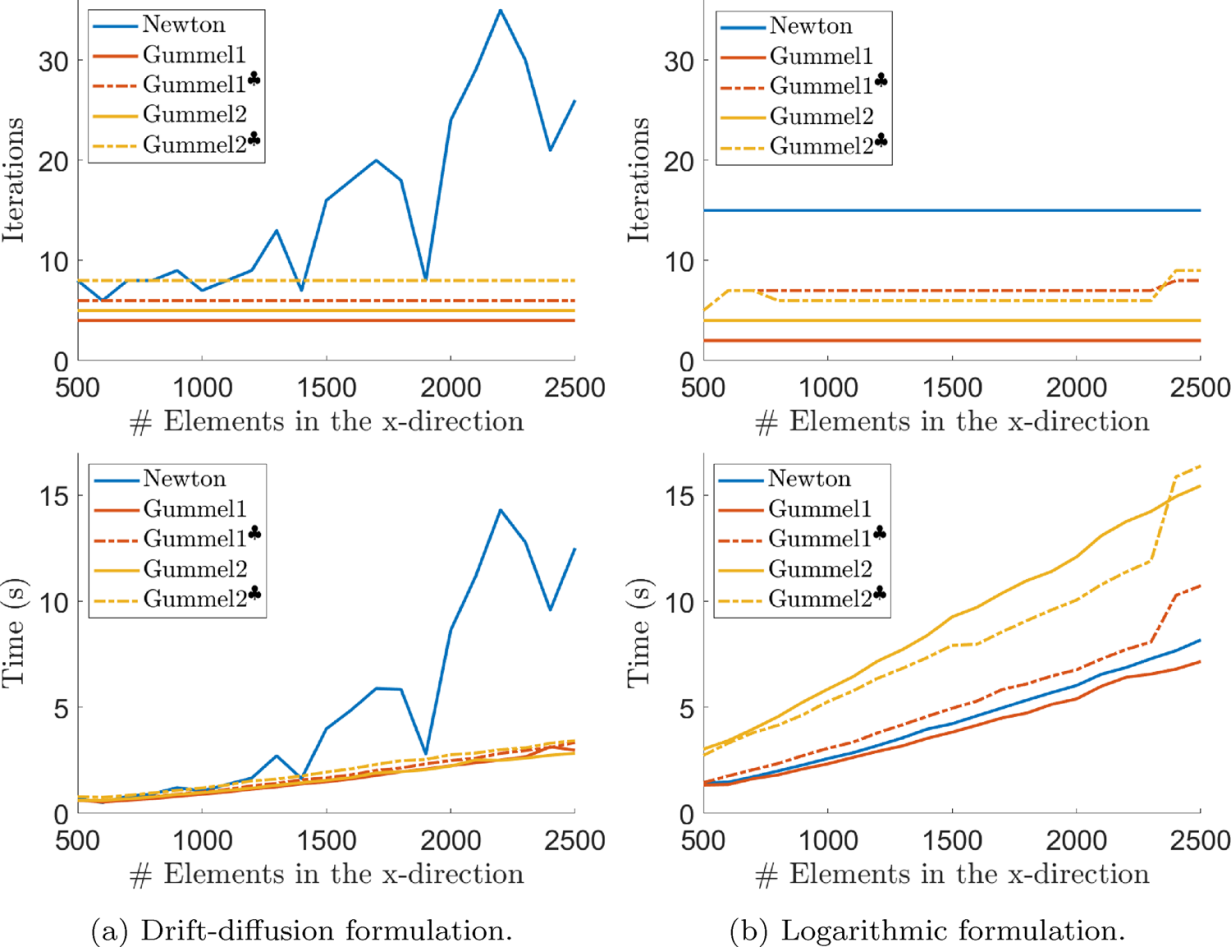
Evolution of the number of iterations (top) and computational time (bottom) with uniform mesh refinement in the *x*-direction, for the solution of the *p*-*n* junction problem with $V_{app}=0$ and with different non-linear solvers

**Table 2:** Number of iterations until convergence, total number of linear system solutions and number of non-zero entries of each system, for both formulations and a mesh with 1500 uniform elements in the *x*-direction

	Drift-diffusion		Logarithmic		
	$\#$Iterations	$\#$Linear systems	$\#$Iterations	$\#$Linear systems	$\#$Non-zero entries
Newton	16	16	15	15	526617
*Gummel1*	4	21 & 9	2	16 & 15	58513 & 234502
*Gummel1* $^{\clubsuit }$	6	16 & 9	7	18 & 17	58513 & 234502
*Gummel2*	5	70	4	66	58513
*Gummel2* $^{\clubsuit }$	8	60	6	57	58513

Figure 6 shows the number of iterations needed to obtain convergence (top) and the total CPU time (bottom) for the solution of the unbiased ($V_{\text {app}}=0$) drift-diffusion (left) and logarithmic (right) models for different meshes under uniform refinement in the *x*-direction, with 4 elements in the vertical axis, and with residual norm and relative error tolerances of $10^{-8}$ and $10^{-6}$, respectively. The CPU time information in Fig. 6 (bottom) is complemented with Table 2, where the required number of linear system solutions, as well as the number of non-zero entries of each system, is reported for both formulations and the particular case of 1500 elements in the *x*-direction. Again, no comparison should be made between the drift-diffusion and the logarithmic formulations since the initial guesses, taken here as properly adjusted $\arctan$-like functions, are not comparable.

In the case of the drift-diffusion formulation, for large problems and low current scenarios, whenever both solvers converge, Gummel approaches are more efficient in terms of computational cost, as can be observed in Fig. 6a and in agreement with the claims in [36]. Furthermore, both versions of Gummel’s method outperform Newton’s method even when intermediate Gummel steps are computed with a relatively large tolerance of $10^{-2}$ (depicted in dashed lines). Even though intermediate relaxed Gummel steps are cheaper, the solver needs more iterations to converge, and the overall computational time is slightly larger (but comparable) to the pristine Gummel approaches. This fact can also be appreciated in Table 2, since the number of linear systems solved using accurate and relaxed intermediate Gummel steps are comparable.

**Fig. 7:**
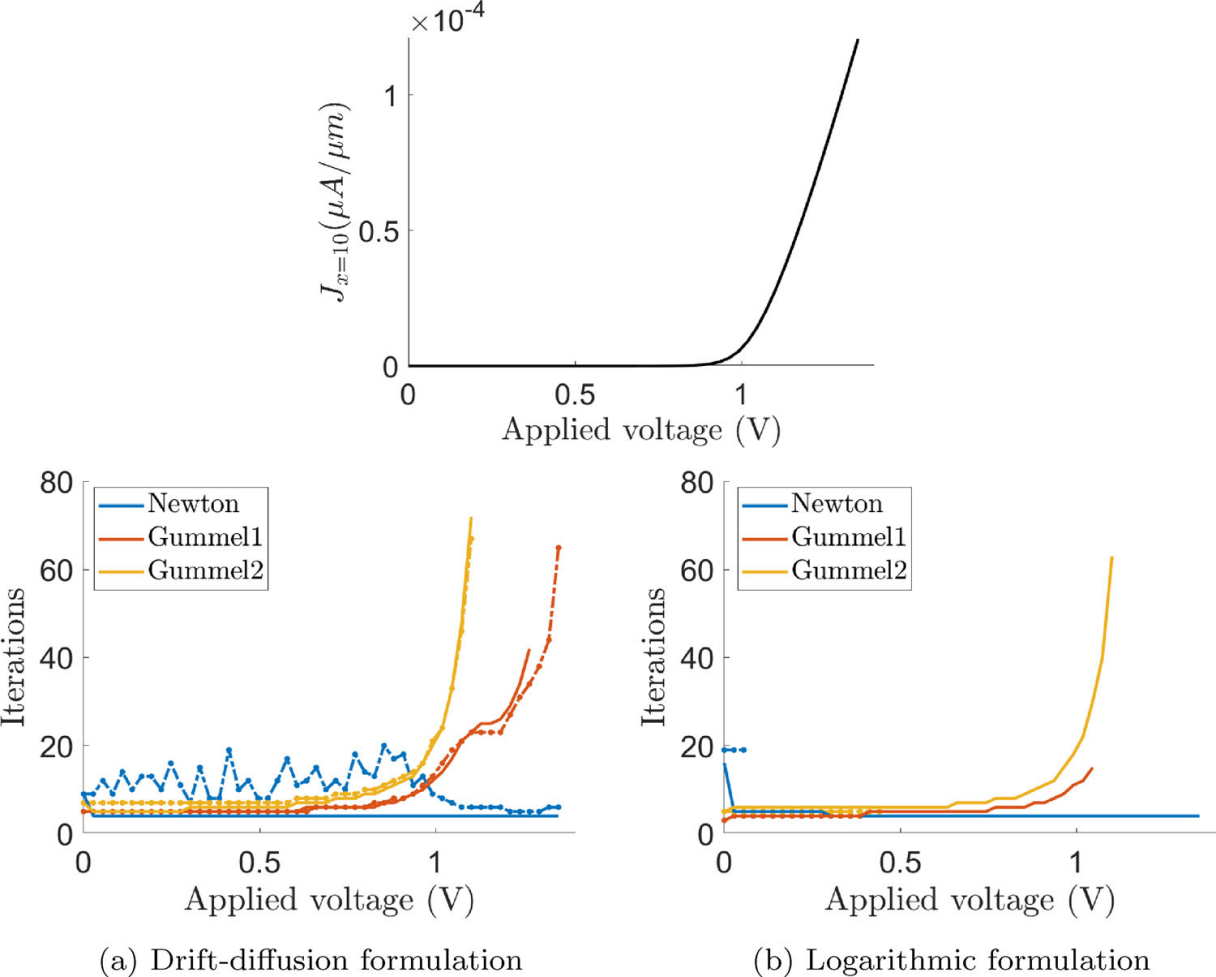
Current–voltage curve of the simulated *p*-*n* junction (top) and evolution of the number of iterations needed for convergence with the applied voltage for the drift-diffusion and logarithmic formulations (bottom). Solid lines refer to the number of iterations required when using a voltage stepping strategy in the applied voltages with voltage increment $\Delta V = 27\,{\textrm{mV}}$, and in dashed lines, the required number of iterations when using $\arctan$-adjusted initial guesses for each of the applied voltages

In the case of the logarithmic formulation, Newton and *Gummel1* methods are the most efficient options, as shown in Fig. 6b. The *Gummel2* approach, that is, replacing the monolithic non-linear system for the charge transport by two decoupled non-linear systems in the third step of the Gummel solver, is not so advantageous for the logarithmic formulation due to the stronger non-linearity of the equations. Similarly to the drift-diffusion formulation, relaxing the tolerance in intermediate Gummel steps increases the number of required iterations to converge, but overall, the required computational time is barely affected.

For large current scenarios (forward biased, $V_{\text {app}}>0$), the performances are very different. Figure 7 (top) shows the current-voltage (J-V) curve of the simulated *p*-*n* junction. The currents reported in the J–V curves are computed as

$$\begin{aligned} \textit{J} = \int _{\Gamma _{\Box }} \varvec{J}\cdot \varvec{\nu }\ dS \ (\mu A /\mu m), \quad \varvec{J}= \varvec{J}_n + \varvec{J}_p, \end{aligned}$$

being $\Gamma _{\Box }$ the surface in contact with the electrode where the current is measured, in this case, $\Gamma _{R}$. It can be seen that the large current regime is attained after a certain threshold voltage around 0.8*V*.

**Fig. 8:**
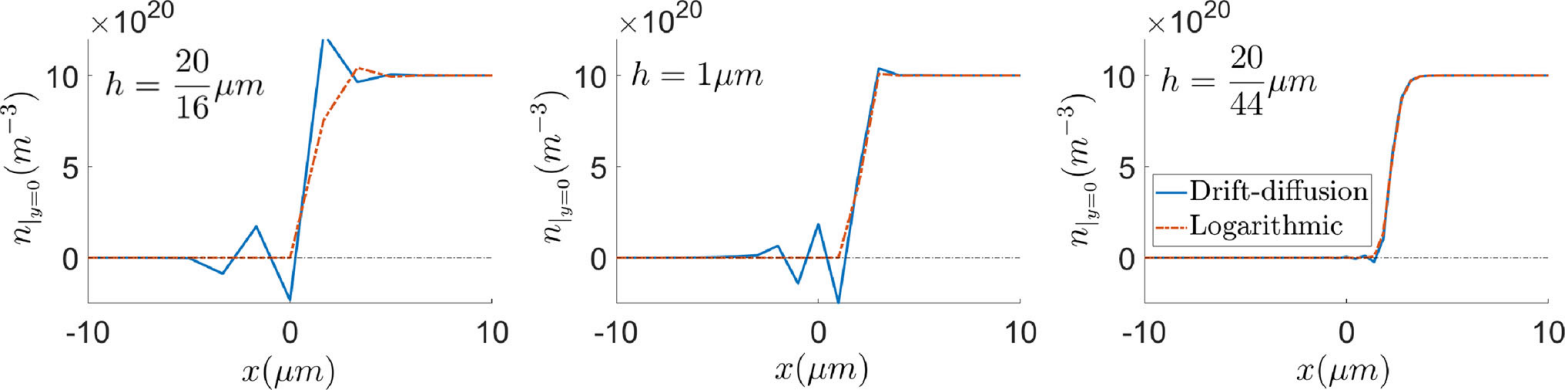
Electron density profile for the drift-diffusion (blue) and the logarithmic (orange) formulations, using $16, \ 20$ and 44 elements in the *x*-direction

**Fig. 9:**
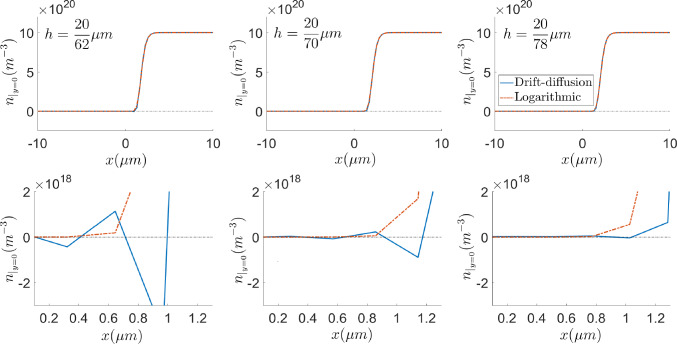
Electron density profile for the drift-diffusion formulation (blue) and the logarithmic formulation (orange), using $62, \ 70$ and 78 elements in the *x*-direction, and a zoom in $[0.1,1.3]\,\upmu {\textrm{m}}$ (bottom)

Figure 7 also shows the plots of the number of iterations needed by each non-linear solver for the drift-diffusion (bottom-left) and the logarithmic (bottom-right) formulations to obtain a converged solution when increasing the applied voltage. Solid lines refer to the number of iterations required when using a voltage stepping strategy in the applied voltages, and in dashed lines, the required number of iterations when using $\arctan$-adjusted initial guesses for each one of the applied voltages.

In this large current regime, for the drift-diffusion formulation, the convergence rate of the Gummel methods is dramatically reduced, in agreement with [1, 36], which constitutes a drawback of this non-linear solver. *Gummel1* and *Gummel2* approaches exhibit a very fast increase of the required number of iterations whereas the Newton strategy is able to converge in few iterations, even with larger values of the applied voltage. For larger values than the ones depicted in the plot, Gummel methods do not even converge (in less than 100 iterations). The use of a voltage stepping strategy with steps of $27\,{\textrm{mV}}$ does not significantly improve the performance of the Gummel solvers whereas it reduces the number of iterations required by the Newton solver.

Regarding the logarithmic formulation, all non-linear solvers fail to converge when not using a voltage stepping strategy, even in low current regime. This behavior is attributed to not close enough initial guesses of the non-linear discretised system. Instead, when a voltage stepping strategy is considered, all the solvers behave properly in low current regime. Once the large current regime is attained, the required number of iterations until convergence blows up for the Gummel solvers, similarly to the drift-diffusion case, whereas the Newton method always converges in few iterations.

Therefore, it can be concluded that Gummel approaches do not perform properly in large current regimes. Consequently, in the remainder of the paper, the Newton solver with a voltage stepping strategy will be considered, unless otherwise explicitly stated.

##### Remark 7

The *p*-*n* junction videos and Fig. 7 have been computed with the alternative homogeneous normal current boundary conditions stated in Appendix B, because they allow the logarithmic formulation to reach larger applied voltage values.

#### Comparison of formulations in terms of mesh requirements

The *stabilisation* effect of the logarithmic formulation in the presence of sharp variations is addressed in this subsection. Figures 8 and 9 show the electron density profile for the drift-diffusion and logarithmic formulations with uniform quadrilateral meshes with 4 elements in the *y*-direction and different element size in the *x*-direction, *h*. It can be appreciated that oscillation-free numerical solutions can be obtained, using coarser meshes, with the logarithmic formulation. While the drift-diffusion formulation still shows spurious oscillations with 44 elements, the logarithmic one does not exhibit them with only 16 elements, as can be observed in the left plot of Fig. 8.

The numerical instabilities in the solution of the drift-diffusion formulation may be reduced by considering finer meshes. However, just zooming in in the layer, one can notice that the spurious oscillations are reduced in amplitude but still persist even with 70 elements. These results are in agreement with [34, 36], where it is stated that the element size must be smaller than the Debye length in order to properly capture the charge fluctuations. Since the sparsity pattern of the Jacobian is roughly the same in both formulations (see Remark 5), the benefit of the logarithmic formulation in terms of computational cost and memory requirement is clear. Oscillation-free solutions can be obtained with the logarithmic formulation solving linear systems with 5733 non-zero entries, while the drift-diffusion formulation needs the solution of linear systems with 27495 non-zero entries. Furthermore, in both Figs. 8 and 9, the plots corresponding to the drift-diffusion formulation contain unsound negative values of the carrier density while, as stated in Sect. 2, the logarithmic formulation always renders positive values.

**Fig. 10:**
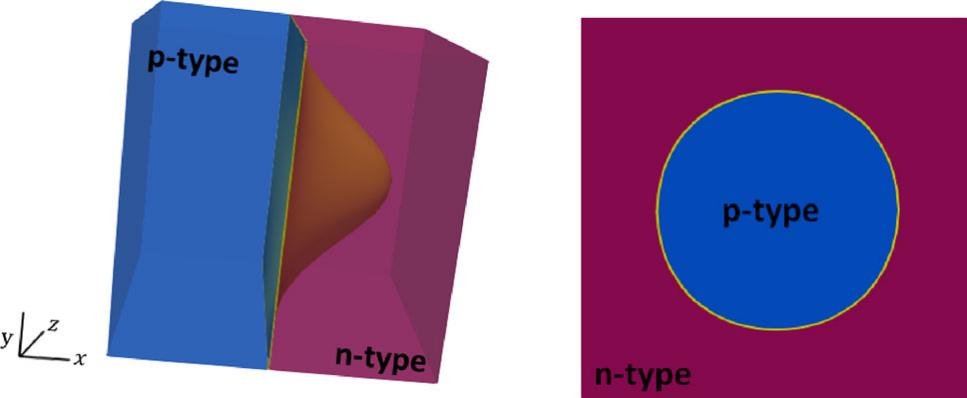
Doping profile of the 3D $p{-}n$ junction device and a cross section at $x=2\,\upmu {\textrm{m}}$. In yellow, the interface shape

A similar behavior can be observed in a 3D *p*-*n* junction device, inspired in the 2D one considered in [33]. A cubic $[-10,10]^3 \,\upmu {\textrm{m}}$ domain with a bi-variate Gaussian-shaped interface between *n*-type and *p*-type materials is considered, as shown in Fig. 10. The interface is defined by the graph $x = f(y,z)$ with

$$\begin{aligned} \displaystyle {f(y,z) = \frac{14}{2}\left( e^{-\frac{y^2}{36}} - e^{-\frac{10^2}{36}}\right) \left( e^{-\frac{z^2}{36}} - e^{-\frac{10^2}{36}}\right) }, \end{aligned}$$

and the boundary conditions and material parameters are the same as in the previous *p*-*n* junction example.

**Fig. 11:**
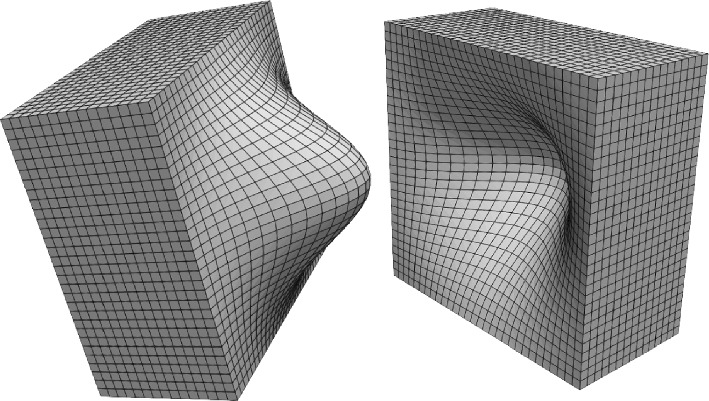
Hexahedral mesh obtained after applying the deformations in (20) to a uniform hexahedral mesh of the cube

Figure 11 illustrates the structure of the hexahedral meshes used for the simulation, which have been built from uniform hexahedral meshes of the cube $[-10,10]^3$, with coordinates (*X*, *Y*, *Z*) that are deformed to (*x*, *y*, *z*) by

20 $$\begin{aligned} x = X + \left( 1+\frac{|X|}{10}\right) f(Y,Z), \ y = Y, \ z =Z. \end{aligned}$$

**Fig. 12:**
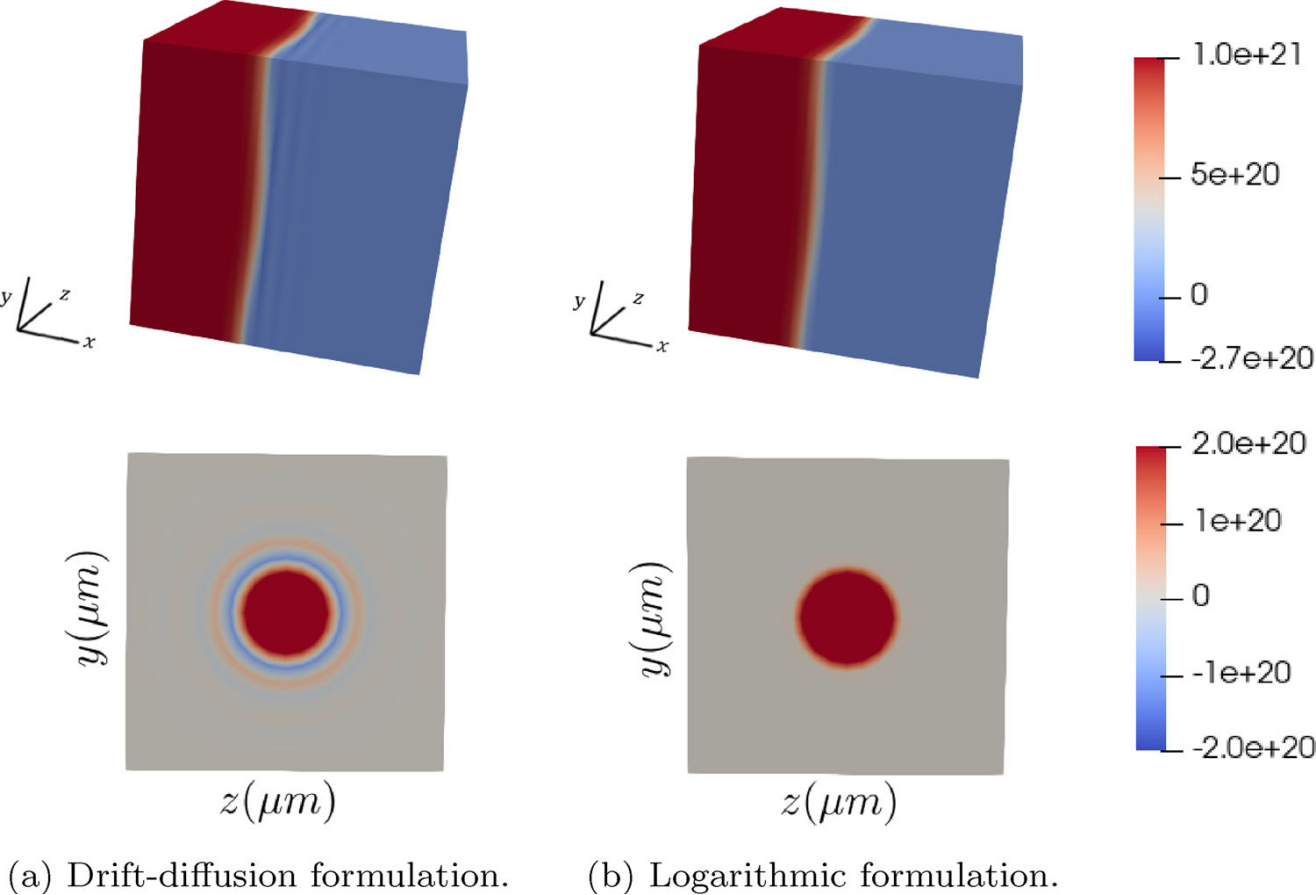
On top, hole concentration for the drift-diffusion (left) and logarithmic (right) formulations, for a mesh of $34 \times 20 \times 20$ elements. At the bottom, the corresponding plots along the cross section $x=2\,\upmu {\textrm{m}}$

Figure 12 (top) depicts the hole concentration with a mesh of $34\times 20 \times 20$ elements. The numerical oscillations exhibited by the drift-diffusion model can already be noticed, fact that is much better appreciated in Fig. 12 (bottom) where the cross sections at $x=2\,\upmu {\textrm{m}}$ are plotted. There, it can be noticed how the logarithmic formulation captures the layer where the hole concentration exhibits a sharp variation while the drift-diffusion formulation produces significant numerical oscillations, pointed out by the consecutive changes in color.

**Fig. 13:**
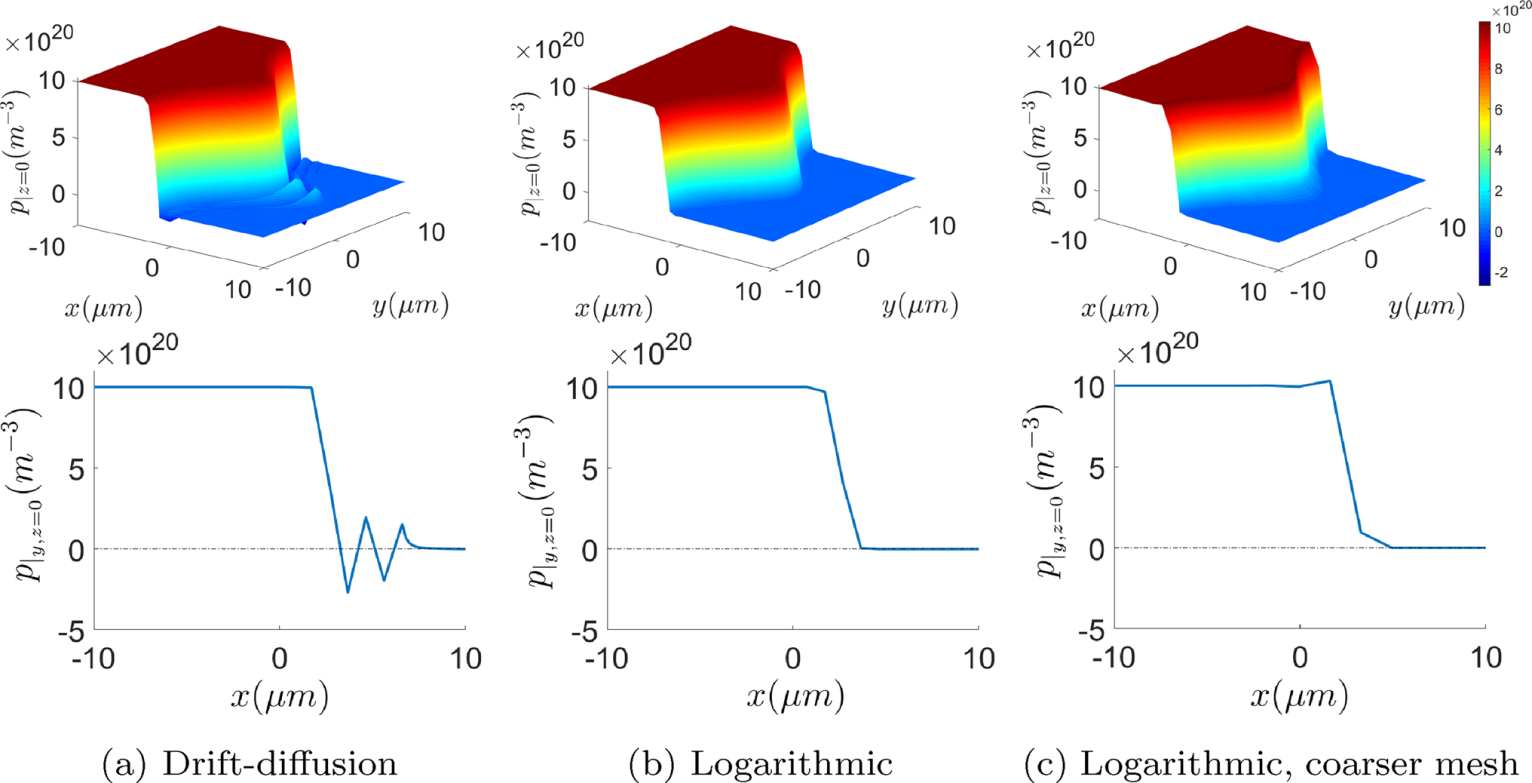
Hole concentration for the drift-diffusion (left) and logarithmic (center) formulations, for a mesh of $34 \times 20 \times 20$ elements, and for the logarithmic formulation with a mesh of $20\times 10 \times 10$ elements (right). On top, the cross sections at $z=0$ and, on the bottom, the cross sections at $y,z=0$

Figure 13 shows the hole concentration in the $z=0\,\upmu {\textrm{m}}$ cross section and the one-dimensional plots at $y,z=0\,\upmu {\textrm{m}}$. Since the junction shape is symmetric with respect to *y* and *z*, same results would be obtained when considering the cross section at $y=0\,\upmu {\textrm{m}}$. The results are in qualitative agreement with the ones obtained in [33], and also evidence the better stability of the logarithmic formulation, which can solve the problem with no instabilities using even coarser meshes, as the one reported in the plot on the right with $20 \times 10 \times 10$ elements.

### MOSFET

**Fig. 14:**
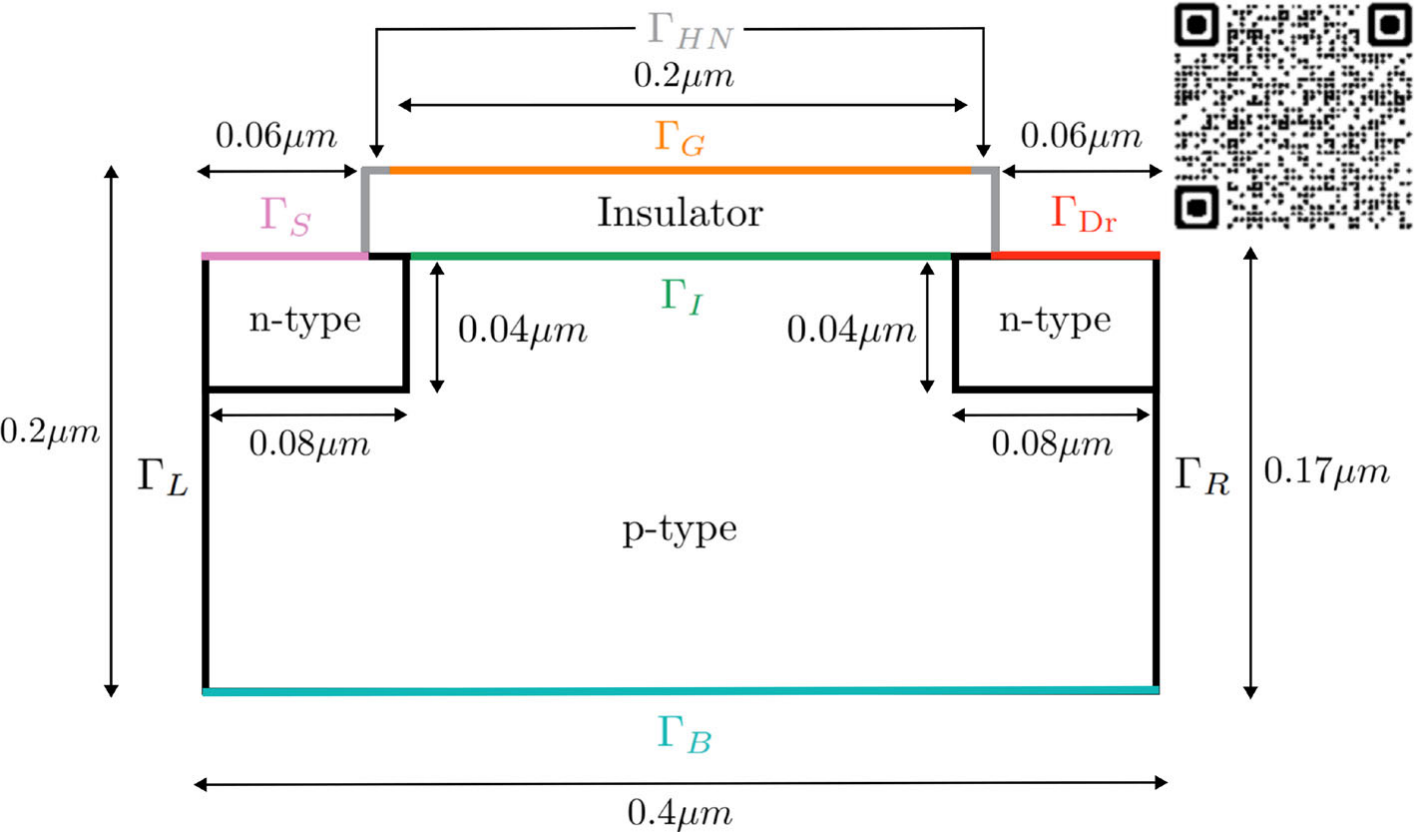
MOSFET material regions and boundary definitions, inspired in the information in [5, 35], and QR code for a video of the creation of the electron channel

As a final example, a physically-realistic two-dimensional device is considered: a MOSFET (Metal Oxide Semiconductor Field Effect Transistor) composed by two small regions of *n*-type silicon, a larger part composed by *p*-type silicon and a thin layer of metal-oxide insulator material, as sketched in Fig. 14. The simulation is performed in the micro scale, with a rectangular semiconductor region of $0.4 \times 0.17 \,\upmu {\textrm{m}}$, a rectangular insulator of $0.28 \times 0.03 \mu m$ on top of the semiconductor, leaving $0.06 \,\upmu {\textrm{m}}$ at each side, and two rectangles of $0.08 \times 0.04 \,\upmu {\textrm{m}}$ representing *n*-type material, overlapping the interface with the insulator region in $0.02\,\upmu {\textrm{m}}$ each. The transport equations are solved only in the semiconductor rectangle, since there are no charge currents in the insulator. In contrast, the Gauss law is solved in the whole domain, insulator and semiconductor materials.

The device works due to differences of potential and, in order to generate them, four ideal Ohmic contacts are placed; one of them at the bottom, called base electrode and identified by $\Gamma _{\text {B}}$, and the other three located at the top boundary of the device. Two of them, called drain and source electrodes, $\Gamma _{\text {Dr}}$ and $\Gamma _{\text {S}}$, respectively, on top of the *n*-type boundary regions and the last one, called gate electrode, on top of the insulator material, occupying $0.2 \,\upmu {\textrm{m}}$, leaving $0.04 \,\upmu {\textrm{m}}$ at each side, identified by $\Gamma _{\text {G}}$.

Regarding the boundary conditions, thermal equilibrium is assumed at contact interfaces, that is, zero net charge, no recombination, and no currents of minor carriers, which is translated in the Dirichlet boundary conditions for carrier densities:

$$\begin{aligned} n = \frac{N_D^+}{2}\left( 1+ \sqrt{1+4\frac{n_i^2}{{N_D^+}^2}}\right) , \quad p = \frac{n_i^2}{n} \quad&\text {at } \Gamma _{\text {Dr}} \ \text {and} \ \Gamma _{\text {S}}, \\ n = \frac{n_i^2}{p}, \quad p = \frac{N_A^-}{2}\left( 1+\sqrt{1+4\frac{n_i^2}{{N_A^-}^2}}\right) \quad&\text {at } \Gamma _{\text {B}}, \end{aligned}$$

and zero normal currents, $\varvec{J}_n \cdot \varvec{\nu }= \varvec{J}_p \cdot \varvec{\nu }= 0$ at $\Gamma _{\text {I}}, \ \Gamma _{\text {L}}$ and $\Gamma _{\text {R}}$.

**Table 3:** Parameters used in the semiconductor simulations

Parameter	Symbol	Synthetic	$p{-}n$ junction	MOSFET	Units
Electric permittivity	$\epsilon$	$1.1 \cdot 10^{-9}$	$6.2\cdot 10^{-10}$	$1.0359\cdot 10^{-10}$	$CV^{-1}m^{-1}$
Electric charge	*q*	$1.60217662 \cdot 10^{-12}$	$1.60217662 \cdot 10^{-19}$	$1.60217662 \cdot 10^{-19}$	*C*
Temperature	*T*	120	300	300	*K*
Lifetimes	$\tau _n,\tau _p$	$5\cdot 10^{-3}$	736	$5 \cdot 10^4$	*ns*
Boltzmann constant	*k*	$1.38064852 \cdot 10^{-11}$	$1.38064852 \cdot 10^{-23}$	$1.38064852 \cdot 10^{-23}$	$m^2 kg s^{-2} K^{-1}$
Band gap energy	$E_{\text {bgap}}$	$1.86253032 \cdot 10^{-8}$	$2.48337376\cdot 10^{-19}$	−	*J*
Conduction density of state	$N_C$	$3.03 \cdot 10^{20}$	$3.97 \cdot 10^{24}$	$3.1 \cdot 10^{25}$	$m^{-3}$
Valence density of state	$N_V$	$3.03 \cdot 10^{20}$	$3.97 \cdot 10^{24}$	$1.8 \cdot 10^{25}$	$m^{-3}$
Donor concentration	$N_D^+$	$2.33 \cdot 10^{18}$	$10^{21}$	$10^{24}$	$m^{-3}$
Acceptor concentration	$N_A^-$	$1.42 \cdot 10^{18}$	$10^{21}$	$10^{23}$	$m^{-3}$
Electron diffusion	$D_n$	1.2	$1.7\cdot 10^{-6}$	$6.85\cdot 10^{-4}$	$m^2s^{-1}$
Hole diffusion	$D_p$	1.3	$1.1\cdot 10^{-6}$	$6.46\cdot 10^{-4}$	$m^2s^{-1}$
Intrinsic carrier concentration	$n_i$	−	−	$10^{16}$	$m^{-3}$

For the electric potential, in the absence of exterior electric field, the device is in equilibrium with the equilibrium voltage

$$\begin{aligned} \phi _{\text {eq}}= &   V_T\sinh ^{-1}\left( \frac{\gamma }{2n_i}\right) \\  = &   \left\{ \begin{array}{ll} \displaystyle {V_T\sinh ^{-1}\left( \frac{N_D^+}{2n_i}\right) } &  \text {at } n\text {-type contacts}\\ \displaystyle {-V_T\sinh ^{-1}\left( \frac{N_A^-}{2n_i}\right) } &  \text {at } p\text {-type contacts.}\\ \end{array} \right. \end{aligned}$$

The difference of potential between regions is modelled by positive applied voltages, $V_{\text {app}}^1$ and $V_{\text {app}}^2$, at contact interfaces as

$$\begin{aligned}&\phi = \phi _{\text {eq}} + V_{\text {app}}^1 \ \text {at } \Gamma _{\text {G}},\\&\phi = \phi _{\text {eq}} + V_{\text {app}}^2 \ \text {at } \Gamma _{\text {S}},\\&\phi = \phi _{\text {eq}} \ \text {at } \Gamma _{\text {Dr}}, \ \Gamma _{\text {B}}, \end{aligned}$$

where the equilibrium potential at the gate contact is assumed to be equal to that of a *p*-type region. Zero normal electric displacement is set in the remaining boundaries of the device, that is $\varvec{D}\cdot \varvec{\nu }= 0$ on $\Gamma _{\text {L}}$, $\Gamma _{\text {R}}$ and $\Gamma _{\text {HN}}$.

Once a positive difference of potential between top and bottom boundaries is created, the holes of the *p*-material close to the insulator film are pushed downwards, accumulating at the bottom part. At the same time, a positive difference of potential between left and right boundaries moves the electrons towards the drain electrode, creating what is known as an electron channel, whose width depends on how large the potential difference is. The material parameters used, extracted from [5, 16], are reported in Table 3.

**Fig. 15:**
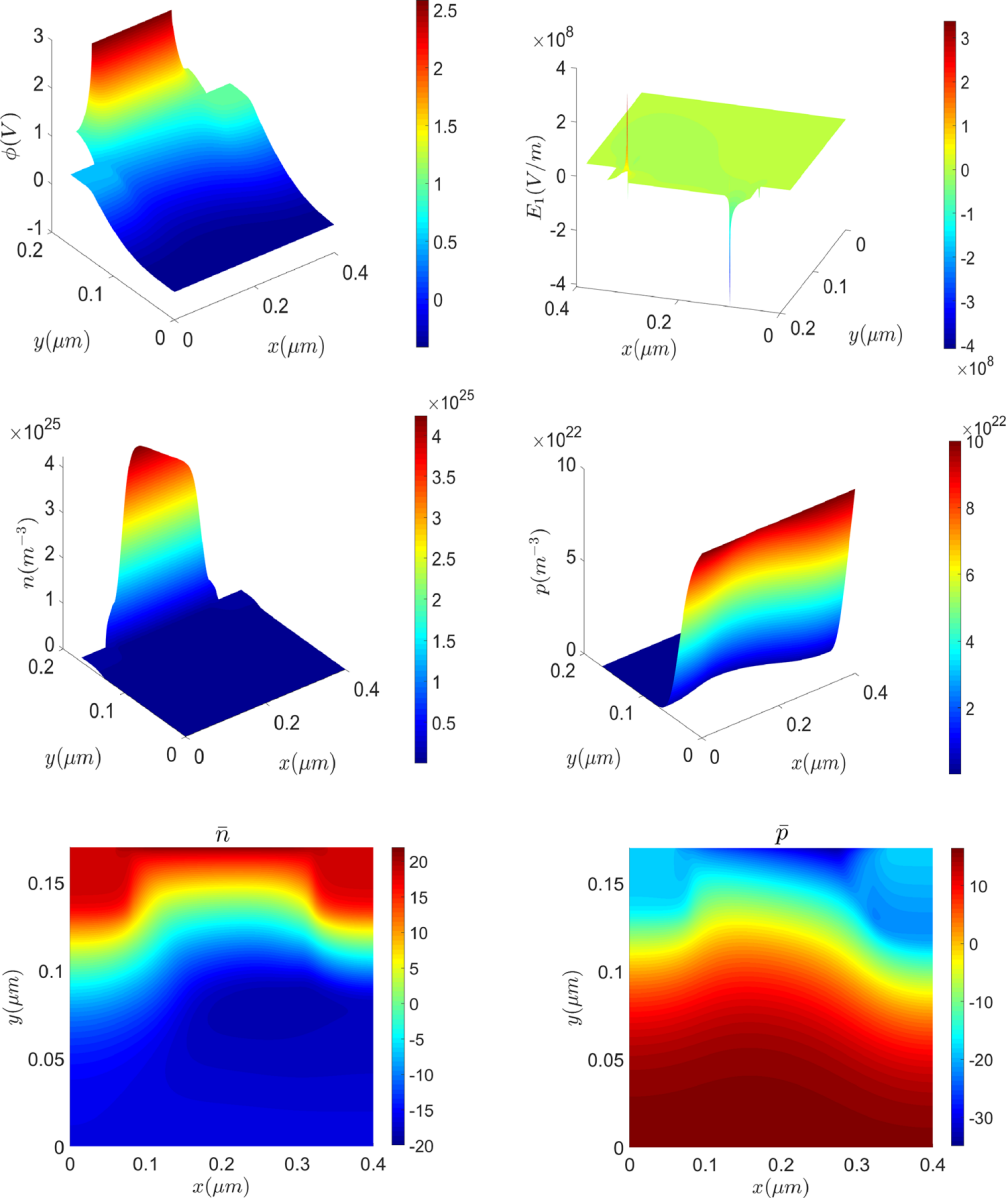
Solution of the MOSFET problem with $V_{\text {app}}^1=3\,{\textrm{V}}, \ V_{\text {app}}^2=0.5\,{\textrm{V}}$: electric potential and *x*-component of the electric field (top), electron and hole densities (mid) and logarithmic version of the electron and hole densities (bottom)

**Fig. 16:**
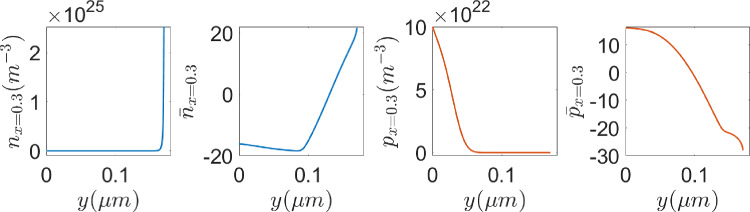
From left to right: cross sections of the electrons density, *n* and ${\bar{n}}$, and the holes density, *p* and ${\bar{p}}$, at $x=0.3\,\upmu {\textrm{m}}$

From a computational viewpoint, it is important noting that the solution of this problem has sharp variations for different reasons. On the one hand, a sharp variation appears across the interface between *n*-type and *p*-type materials, similarly to the *p*-*n* junction. On the other hand, sharp variations are also induced due to the creation of the electron channel, where electrons accumulate close to the insulator interface and holes do so around the base contact. Furthermore, singularities are also created in the electric field. This features can be observed in Fig. 15, which shows the solution of the problem, obtained solving the logarithmic formulation on a uniform, quadrilateral mesh with $640\times 544$ elements in the semiconductor material and $384 \times 96$ elements in the insulator region.

**Fig. 17:**
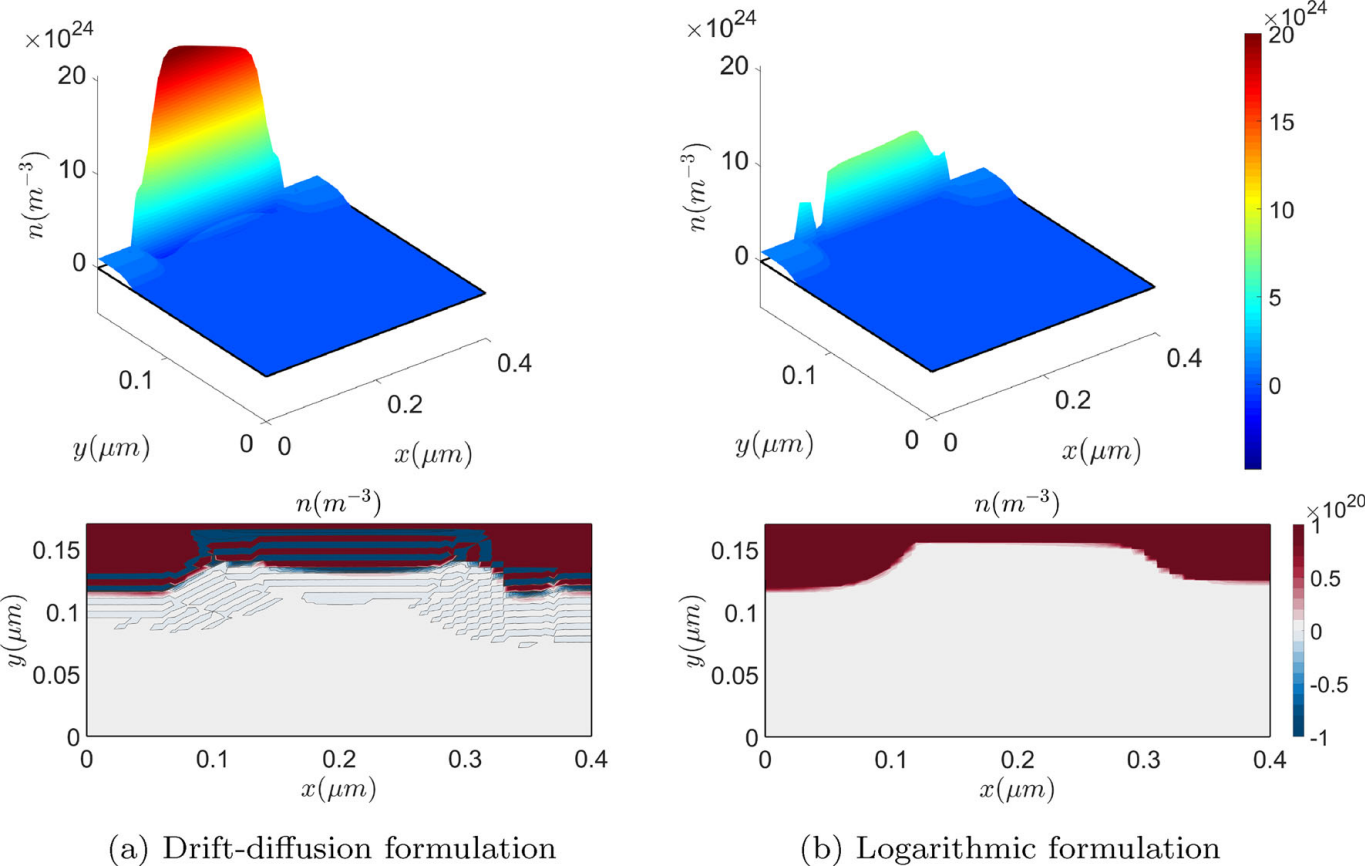
On top, electron’s density obtained with the drift-diffusion (left) and the logarithmic (right) formulations on a very coarse mesh. On the bottom, the corresponding two-dimensional plots, with a contour plot of 7 equispaced values in $[-8 \cdot 10^{-19},-2\cdot 10^{12}]$

**Fig. 18:**
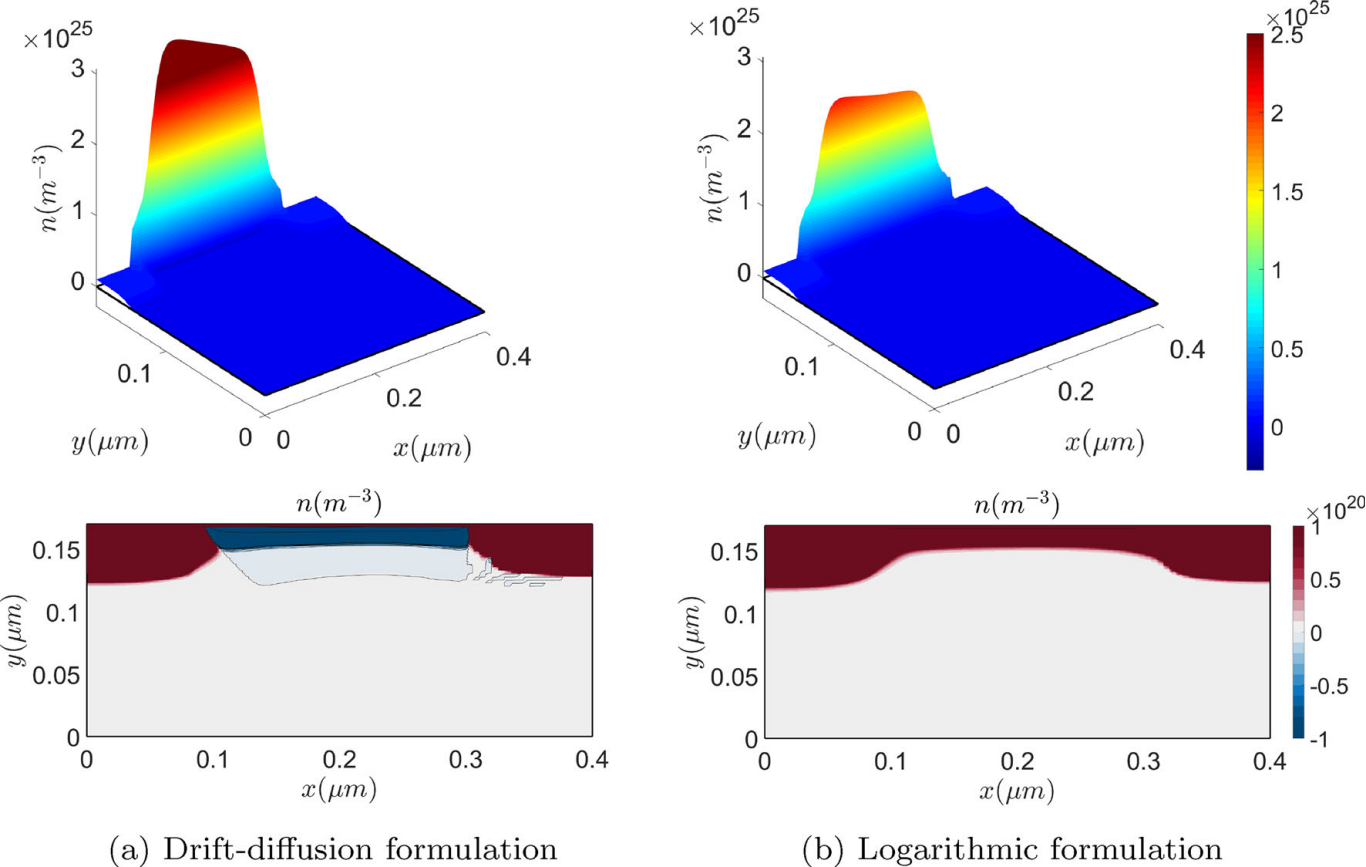
On top, electron’s density obtained with the drift-diffusion (left) and the logarithmic (right) formulations on a coarse mesh (top). On the bottom, the corresponding two dimensional plots, with a contour plot of 7 equispaced values in $[-8 \cdot 10^{-19},-2\cdot 10^{12}]$

The results are in qualitative agreement with the MOSFET device in [5], which considers electric-field-dependent carrier mobilities. The creation of the channel of electrons when increasing the differences of electric potential between regions can be seen at [25] or scanning the QR code at Fig. 14.

**Fig. 19:**
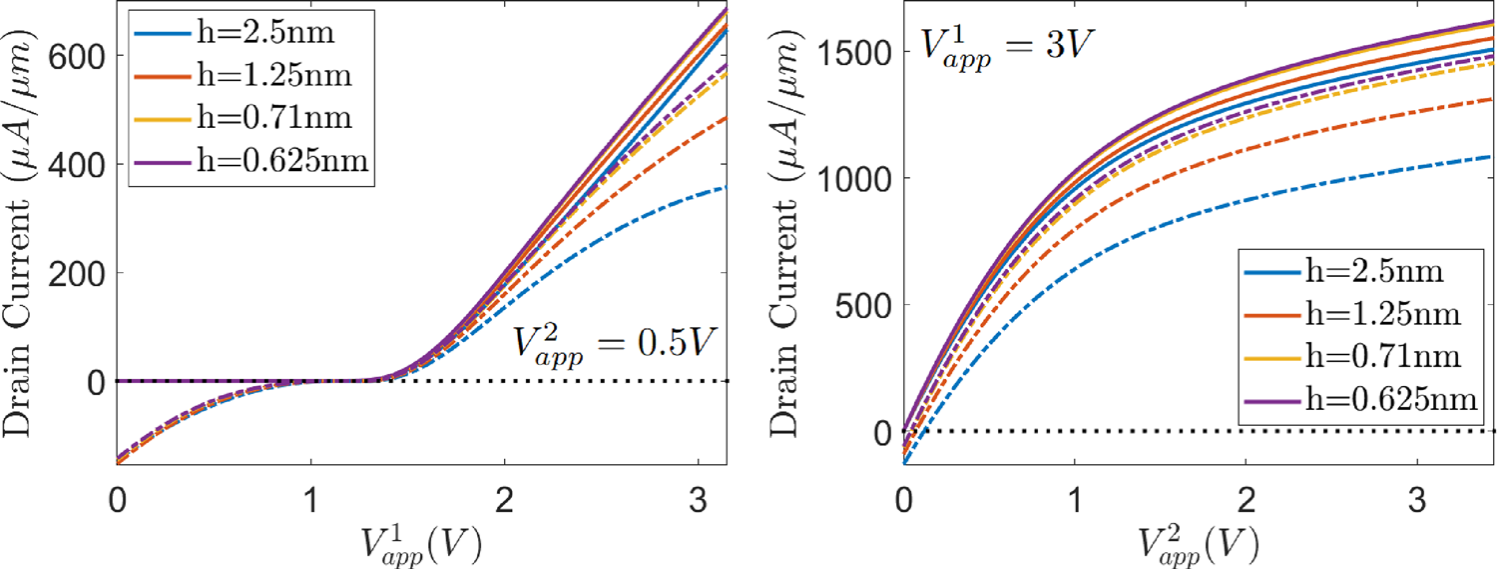
Evolution of the drain current with the gate voltage for a fixed drain voltage with $V_{\text {app}}^2 = 0.5\,{\textrm{V}}$ (left) and evolution of the drain current with the drain voltage for a fixed gate voltage with $V_{\text {app}}^1=3\,{\textrm{V}}$ (right) for different meshes with element size *h*. Results for the drift-diffusion and logarithmic formulations are plotted with dashed and continuous lines, respectively

Focusing on Fig. 16, where cross sections of *n*, ${\bar{n}}$, *p* and ${\bar{p}}$ are reported, one can observe that the sharp layers of the drift-diffusion set up carrier densities are much smoother in their dimensionless logarithmic counterparts. Therefore, oscillation-free solutions are expected to be obtained with coarser meshes using the logarithmic formulation. To confirm the conclusions in the previous Section on the mesh requirements, the problem is solved in a very coarse uniform, quadrilateral mesh with $40\times 34$ elements in the semiconductor material and $28\times 6$ elements in the insulator region. Figure 17 (top) shows the electron density numerical solution on the very coarse mesh with the drift-diffusion model (left) and the logarithmic model (right).

The results are not accurate in any case, but one can appreciate in the plots at the bottom of the Figure that the logarithmic formulation provides an oscillation-free solution, in contrast with the drift-diffusion formulation, which, furthermore, exhibits large non-physical negative values.

Using a slightly finer mesh ($80\times 68$ elements in the semiconductor material and $56\times 12$ elements in the insulator region) can seem to overcome both, the spurious oscillations and the accuracy problems, looking at the results plotted in Fig. 18 (top). However, paying attention at the corresponding plots in the bottom of the Figure, one can notice that the drift-diffusion formulation still renders numerical oscillations and, consequently, it has physically unsound negative concentrations.

The benefits of the logarithmic formulation in the presence of sharp variations can be further checked with the current-voltage (J-V) characteristic curves, shown in Fig. 19 for different uniform, quadrilateral meshes under uniform refinement, with characteristic element sizes $h=2.5, 1.25, 0.71, 0.625$nm. On the left, the curves depict the drain current obtained when prescribing the drain voltage with $V_{\text {app}}^2=0.5V$ and varying the gate voltage from 1*V* to 2.5*V*. There, it can be clearly appreciated that from a threshold voltage of about 1*V* on, the electron channel is created, allowing the generation of drain current. On the right, the curves show the influence of the drain voltage in the drain current when prescribing the gate voltage with $V_{\text {app}}^1 = 3V$. There, it can be appreciated how, whenever a difference of potential between source and drain regions is imposed, an electron channel is created yielding effective drain current, whose intensity saturates when the drain voltage increases.

Regarding the numerical solutions, the one obtained with the drift-diffusion formulation presents a strong dependence on mesh resolution, whereas the J-V curve obtained with the logarithmic formulation is not so sensitive to mesh size. Most likely, this is due to the ability of logarithmic formulation of computing sharp variations with no spurious oscillations. Furthermore, in this particular example, the non-linear solver is more robust in combination with the logarithmic formulation. The problem is solved using a voltage stepping strategy in applied voltages. However, the first step in the logarithmic formulation can be solved considering an initial guess defined as zeros on the bulk and Neumann face nodes, and the corresponding Dirichlet values on Dirichlet face nodes, whereas the drift-diffusion formulation fails to converge with the same initial guess. To obtain a solution for the drift-diffusion formulation, in addition to the increments in the applied voltages, an incremental strategy in mobilities, from 0 to the ones reported in Table 3, has also been implemented. Thus, in this application the logarithmic formulation is significantly more efficient, because it does not require as many incremental steps for the non-linear solution, and coarser meshes can be used to approximate the solution, further reducing the computational cost.

## Conclusions

In this work, the semiconductor modelling equations are revisited, providing an alternative formulation in terms of dimensionless logarithmic variables that ensures the positiveness of the carrier densities, according to their physical meaning. The numerical experiments with 2D and 3D p-n junctions, and a MOSFET device, point out the applicability of the logarithmic formulation, demonstrating that accurate oscillation-free solutions can be achieved with coarser meshes than the ones needed in the drift-diffusion problem.

In addition, Gummel’s method is adapted to the logarithmic formulation, and its performance is numerically tested and compared with a monolithic Newton-Raphson solver.

A p-n junction is considered to assess the robustness with respect to the initial guess, concluding that Newton’s method exhibits more robustness for the drift-diffusion formulation, and both solvers have similar behaviour for the logarithmic one.

On the other hand, Gummel’s method can reach convergence with significantly lower computational cost than Newton-Raphson, but only for the drift-diffusion formulation and low current regimes. In large current scenarios, which are the most interesting situations to be simulated, the rate of convergence of Gummel’s method is dramatically reduced in both formulations, and it fails to converge for large applied voltage, hampering its practical applicability.

To conclude, the logarithmic formulation together with a Newton-Raphson solver seems to be the most efficient and robust choice for realistic applications, whose applicability has been demonstrated with a MOSFET and a 3D p-n junction simulations.

Finally, it is worth mentioning that the ideas of this manuscript can also be applied for the extension of the logarithmic formulation to hydrodynamic models, which are known to reproduce better the physics than the Van-Roosbroeck equations at sub-micron scales.

## Data Availability

Data sharing is not applicable to this article as no datasets were generated or analysed during the current study. The information to reproduce the numerical results is included in the paper.

## Appendix: Gummel solver

This appendix states in detail the intermediate problems to be solved for each different strategy in the third step of the Gummel solver, to obtain approximations $n^{k+1}$ and $p^{k+1}$ (or ${\bar{n}}^{k+1}$ and ${\bar{p}}^{k+1}$). The potential $\phi ^{k+1}$ (or $\bar{\phi }^{k+1}$) is assumed to be already computed in the third step, and $n^k, \ p^k$ (or ${\bar{n}}^k,\ {\bar{p}}^k$) are also already computed in the previous Gummel iteration.

### Drift-diffusion formulation

In the drift-diffusion formulation, *Gummel1* corresponds to a monolithic Newton-Raphson solution of the discretisation, in a Finite Element space, of the coupled non-linear system in $n^{k+1}$ and $p^{k+1}$, (15b) and (15c). That is,

$$\begin{aligned}&D_n d(\zeta ,n^{k+1}) - \mu _n c(\zeta ,\phi ^{k+1},n^{k+1})\\&+ m(\zeta ,R(n^{k+1},p^{k+1})) = \frac{1}{q}r_n(\zeta )\\&D_p d(\eta ,p^{k+1}) + \mu _p c(\eta ,\phi ^{k+1},p^{k+1})\\&+ m(\eta ,R(n^{k+1},p^{k+1})) = \frac{1}{q}r_p(\eta ). \end{aligned}$$

The label *Gummel2*, corresponds to two Newton-Raphson solutions of two non-linear systems in $n^{k+1}$ and $p^{k+1}$, using values $p^k$ and $n^k$ from the previous Gummel iteration, respectively. That is,

$$\begin{aligned}&D_n d(\zeta ,n^{k+1}) - \mu _n c(\zeta ,\phi ^{k+1},n^{k+1}) \\&+ m(\zeta ,R(n^{k+1},p^{k})) = \frac{1}{q}r_n(\zeta )\\&D_p d(\eta ,p^{k+1}) + \mu _p c(\eta ,\phi ^{k+1},p^{k+1}) \\&+ m(\eta ,R(n^{k},p^{k+1})) = \frac{1}{q}r_p(\eta ). \end{aligned}$$

Note that the systems are non-linear only due to the recombination term.

Finally, *Gummel3* corresponds to the solution of two linear systems in $n^{k+1}$ and $p^{k+1}$, using values $p^k$ and $n^k$ from the previous Gummel iteration in the recombination terms of both equations. That is,

$$\begin{aligned}&D_n d(\zeta ,n^{k+1}) - \mu _n c(\zeta ,\phi ^{k+1},n^{k+1})\\&+ m(\zeta ,R(n^{k},p^{k})) = \frac{1}{q}r_n(\zeta )\\&D_p d(\eta ,p^{k+1}) + \mu _p c(\eta ,\phi ^{k+1},p^{k+1}) \\&+ m(\eta ,R(n^{k},p^{k})) = \frac{1}{q}r_p(\eta ). \end{aligned}$$

### Logarithmic formulation

Similarly, for the logarithmic formulation, *Gummel1* stands for the monolithic Newton-Raphson solution of the coupled non-linear system in ${\bar{n}}^{k+1}$ and ${\bar{p}}^{k+1}$, (16b) and (16c)

$$\begin{aligned}&d(\zeta ,{\bar{n}}^{k+1}-\bar{\phi }^{k+1}) - c({\bar{n}}^{k+1},{\bar{n}}^{k+1}-\bar{\phi }^{k+1},\zeta ) \\&+m(\zeta ,{\bar{R}}_n({\bar{n}}^{k+1},{\bar{p}}^{k+1})) = \frac{1}{q}r_{{\bar{n}}}(\zeta )\\&d(\eta ,{\bar{p}}^{k+1}+\bar{\phi }^{k+1}) - c({\bar{p}}^{k+1},{\bar{p}}^{k+1}+\bar{\phi }^{k+1},\zeta )\\&+ m(\eta ,{\bar{R}}_p({\bar{n}}^{k+1},{\bar{p}}^{k+1})) = \frac{1}{q}r_{{\bar{p}}}(\eta ) . \end{aligned}$$

The label *Gummel2* corresponds to two Newton-Raphson solutions of the two non-linear systems, depending on ${\bar{n}}^{k+1}$ and ${\bar{p}}^{k+1}$, respectively:

$$\begin{aligned}&d(\zeta ,{\bar{n}}^{k+1}-\bar{\phi }^{k+1}) - c({\bar{n}}^{k+1},{\bar{n}}^{k+1}-\bar{\phi }^{k+1},\zeta )\\&+ m(\zeta ,{\bar{R}}_n({\bar{n}}^{k+1},{\bar{p}}^{k})) = \frac{1}{q}r_{{\bar{n}}}(\zeta )\\&d(\eta ,{\bar{p}}^{k+1}+\bar{\phi }^{k+1}) - c({\bar{p}}^{k+1},{\bar{p}}^{k+1}+\bar{\phi }^{k+1},\eta ) \\&+ m(\eta ,{\bar{R}}_p({\bar{n}}^{k},{\bar{p}}^{k+1})) = \frac{1}{q}r_{{\bar{p}}}(\eta ) . \end{aligned}$$

Finally, the *Gummel3* strategy, solves two linear systems on ${\bar{n}}^{k+1}$ and ${\bar{p}}^{k+1}$, respectively:

$$\begin{aligned}&d(\zeta ,{\bar{n}}^{k+1}-\bar{\phi }^{k+1}) - c({\bar{n}}^{k},{\bar{n}}^{k+1}-\bar{\phi }^{k+1},\zeta ) \nonumber \\&+ m(\zeta ,{\bar{R}}_n({\bar{n}}^{k},{\bar{p}}^{k})) = \frac{1}{q}r_{{\bar{n}}}(\zeta )\nonumber \\&d(\eta ,{\bar{p}}^{k+1}+\bar{\phi }^{k+1}) - c({\bar{p}}^{k},{\bar{p}}^{k+1}+\bar{\phi }^{k+1},\eta ) \nonumber \\&+ m(\eta ,{\bar{R}}_p({\bar{n}}^{k},{\bar{p}}^{k})) = \frac{1}{q}r_{{\bar{p}}}(\eta ) . \end{aligned}$$

## Appendix: charge neutrality conditions

If the boundaries are sufficiently far from the interface, charge neutrality boundary conditions can be stated as

21 $$\begin{aligned}  &   n=\frac{\gamma }{2}\left( 1+\sqrt{1+4\frac{n_i^2}{\gamma ^2}}\right) , \quad p=\frac{n_i^2}{n}, \end{aligned}$$

for n-type material, and

22 $$\begin{aligned}  &   p=-\frac{\gamma }{2}\left( 1+\sqrt{1+4\frac{n_i^2}{\gamma ^2}}\right) , \quad n=\frac{n_i^2}{p}, \end{aligned}$$

for p-type material. In the case of the *p*-*n* junction, the second condition in (21) and (22), for *p* and *n*, respectively, can be replaced by an homogeneous normal current condition:

23 $$\begin{aligned} J_p \cdot \varvec{\nu }= 0 \ \text {for} \ x=-L, \quad J_n \cdot \varvec{\nu }= 0 \ \text {for} \ x=L , \end{aligned}$$

since (23) together with the two first conditions in (21) and (22) imply the two second conditions.

Indeed, looking at Fig. 4, one can notice that locally near the left boundary, the electric potential $\phi$ is constant, and therefore, since it is a one-dimensional problem, one can approximate ${\partial p}/{\partial x}\simeq J_p \cdot \varvec{\nu }=0$, which implies that *p* is almost constant around $x=-L$. To determine the constant, since $\Delta \phi \simeq 0$ close to the boundary, one can deduce from the Gauss law that $p-n+\gamma \simeq 0$. Thus,

$$\begin{aligned} p\simeq n-\gamma =\frac{\gamma }{2}\left( -1+\sqrt{1+4\frac{n_i^2}{\gamma ^2}}\right) \end{aligned}$$

and, therefore, it can be checked that $np\simeq n_i^2$ as stated in (21). Analogously, one can justify that imposing $J_n \cdot \varvec{\nu }= 0$ on $x=L$ also implies $np\simeq n_i^2$.

## References

[1] Bank R, Coughran W, Driscoll M, Smith R, Fichtner W (1989) Iterative methods in semiconductor device simulation. Comput Phys Commun 53(1):201–212. 10.1016/0010-4655(89)90160-4

[2] Barnes JJ, Lomax RJ (1977) Finite-element methods in semiconductor device simulation. IEEE Trans Electron Devices 24(8):1082–1089. 10.1109/T-ED.1977.18880

[3] Brezzi F, Marini L, Micheletti S, Pietra P, Sacco R, Wang S (2005) Discretization of semiconductor device problems (I). In: Handbook of numerical analysis, vol 13. Elsevier. 10.1016/S1570-8659(04)13004-4

[4] Chen G, Monk P, Zhang Y (2019) An hdg method for time-dependent drift-diffusion model of semiconductor devices. J Sci Comput 20:420–443. 10.48550/ARXIV.1811.09705

[5] Chen L, Bagci H (2020) Steady-state simulation of semiconductor devices using discontinuous Galerkin methods. IEEE Access 8:16203–16215. 10.1109/ACCESS.2020.2967125

[6] Chen R, Liu J (2003) Monotone iterative methods for the adaptive finite element solution of semiconductor equations. J Comput Appl Math 159(2):341–364. 10.1016/S0377-0427(03)00538-7

[7] Cummings DJ, Law ME, Cea S, Linton T (2009) Comparison of discretization methods for device simulation. In: 2009 International conference on simulation of semiconductor processes and devices, pp 1–4. 10.1109/SISPAD.2009.5290236

[8] Deinega A, John S (2012) Finite difference discretization of semiconductor drift-diffusion equations for nanowire solar cells. Comput Phys Commun 183(10):2128–2135. 10.1016/j.cpc.2012.05.016

[9] Entner R (2007) Modeling and simulation of negative bias temperature instability. Ph.D. thesis, Fakultär Elektrotechnik und Informationstechnik. Technische Universität Wien. 10.34726/hss.2007.10123

[10] Franz AF, Franz GA, Selberherr S, Ringhofer C, Markowich P (1983) Finite boxes-a generalization of the finite-difference method suitable for semiconductor device simulation. IEEE Trans Electron Devices 30(9):1070–1082. 10.1109/T-ED.1983.21261

[11] Grasser T, Tang TW, Kosina H, Selberherr S (2003) A review of hydrodynamic and energy-transport models for semiconductor device simulation. Proc IEEE 91(2):251–274. 10.1109/JPROC.2002.808150

[12] Gummel H (1964) A self-consistent iterative scheme for one-dimensional steady state transistor calculations. IEEE Trans Electron Devices 11(10):455–465. 10.1109/T-ED.1964.15364

[13] Hall R (1952) Electron-hole recombination in germanium. Phys Rev 87:387–387. 10.1103/PhysRev.87.387

[14] Hecht F, Marrocco A, Caquot E, Filoche M (1991) Semiconductor device modeling for heterojunctions structures with mixed finite elements. Int J Comput Math Electr Electron Eng (COMPEL) 10(4):425–438. 10.1108/eb051718

[15] Kumar G, Singh M, Ray A, Trivedi G (2017) An fem based framework to simulate semiconductor devices using streamline upwind Petrov–Galerkin stabilization technique. In: 2017 27th International conference Radioelektronika (ELEKTRONIKA), pp 1–5. 10.1109/RADIOELEK.2017.7936644

[16] Levinshtein M, Rumyantsev S, Shur M (1996) Handbook series on semiconductor parameters. World Scientific, Singapore. 10.1142/2046

[17] Li Y, Chen P, Liu J, Chao T, Sze" S (2000) Adaptive finite volume simulation of semiconductor devices on cluster architecture, pp 107–112. World Scientific and Engineering Academy and Society, Greece. 10.13140/2.1.2878.5287

[18] Liu Y, Shu C (2016) Analysis of the local discontinuous Galerkin method for the drift-diffusion model of semiconductor devices. Sci China Math 59:115–140. 10.1007/s11425-015-5055-8

[19] Machek J, Selberherr S (1983) A novel finite-element approach to device modeling. IEEE Trans Electron Devices 30(9):1083–1092. 10.1109/T-ED.1983.21262

[20] Markowich PA (1985) A finite difference method for the basic stationary semiconductor device equations, pp 285–301. Birkhäuser Boston, Boston. 10.1007/978-1-4612-5160-6_17

[21] Micheletti S (2001) Stabilized finite elements for semiconductor device simulation. Comput Vis Sci 3:177–183. 10.1007/s007910000046

[22] Miller J, Schilders W, Wang S (1999) Application of finite element methods to the simulation of semiconductor devices. Rep Prog Phys 62(3):277. 10.1088/0034-4885/62/3/001

[23] Nanz G (1991) A critical study of boundary conditions in device simulation. In: Simulation of semiconductor devices and processes, vol 4. https://in4.iue.tuwien.ac.at/pdfs/sisdep1991/pdfs/Nanz_33.pdf

[24] Polak SJ, Heijer CD, Schilders WHA, Markowich P (1987) Semiconductor device modelling from the numerical point of view. Int J Numer Meth Eng 24(4):763–838. 10.1002/nme.1620240408

[25] Pérez-Escudero S (2024) Creation of the electron channel in an n-mosfet. www.youtube.com/watch?v=HvvYc6aC6cY

[26] Pérez-Escudero S (2024) p-n junction in forward bias regime. www.youtube.com/watch?v=JE42VygF7EE

[27] Pérez-Escudero S (2024) p-n junction in reverse bias regime. www.youtube.com/watch?v=aBETTHyuM2g

[28] Quarteroni A, Valli A (2008) Numerical approximation of partial differential equations. Springer, Berlin. 10.1007/978-3-540-85268-1

[29] Roosbroeck WV (1950) Theory of the flow of electrons and holes in germanium and other semiconductors. Bell Syst Tech J 29(4):560–607. 10.1002/j.1538-7305.1950.tb03653.x

[30] Rupp K, Bina M, Wimmer Y, Jungel A, Crasser T (2014) Cell-centered finite volume schemes for semiconductor device simulation. In: 2014 International conference on simulation of semiconductor processes and devices (SISPAD), pp 365–368. 10.1109/SISPAD.2014.6931639

[31] Scharfetter DL, Gummel HK (1969) Large-signal analysis of a silicon read diode oscillator. IEEE Trans Electron Devices 16(1):64–77. 10.1109/T-ED.1969.16566

[32] Shockley W, Read WT (1952) Statistics of the recombinations of holes and electrons. J Phys Rev 87:835–842. 10.1103/PhysRev.87.835

[33] Simpson R, Bordas S, Asenov A, Brown A (2012) Enriched residual free bubbles for semiconductor device simulation. Comput Mech 50:119–133. 10.1007/s00466-011-0658-6

[34] Stamatopoulos P, Zeneli M, Nikolopoulos A, Bellucci A, Trucchi DM, Nikolopoulos N (2021) Introducing a 1d numerical model for the simulation of pn junctions of varying spectral material properties and operating conditions. Energy Convers Manag 230:113819. 10.1016/j.enconman.2020.113819

[35] Szuhàr M (1981) Two-dimensional MOS transistor simulation. KFKI-Reports. http://real-eod.mtak.hu/id/eprint/7359

[36] Vasileska D, Goodnick S, Klimeck G (2010) Computational electronics: semiclassical and quantum device modeling and simulation. CRC Press, London. 10.1201/b13776

